# Proton Conductive Zr-Phosphonate UPG-1—Aminoacid Insertion as Proton Carrier Stabilizer

**DOI:** 10.3390/molecules25153519

**Published:** 2020-07-31

**Authors:** Sérgio M. F. Vilela, Pablo Salcedo-Abraira, Alejandro Gómez-Peña, Philippe Trens, Alejandro Várez, Fabrice Salles, Patricia Horcajada

**Affiliations:** 1 Advanced Porous Materials Unit (APMU), IMDEA Energy, Avda. Ramón de la Sagra 3, E-28935 Móstoles, Madrid, Spain; smfvilela@gmail.com (S.M.F.V.); pablo.salcedo@imdea.org (P.S.-A.); alejandro.gomez186@gmail.com (A.G.-P.); 2 ICGM, Univ. Montpellier, CNRS, ENSCM, 34095 Montpellier, France; philippe.trens@enscm.fr; 3 Department of Materials Science and Engineering and Chemical Engineering, Universidad Carlos III de Madrid, Avda. Universidad 30, E-28911 Leganés, Madrid, Spain

**Keywords:** Metal-Organic Frameworks, proton carriers, lysine, ion conductivity

## Abstract

Proton exchange membrane fuel cells (PEMFCs) are an attractive green technology for energy generation. The poor stability and performances under working conditions of the current electrolytes are their major drawbacks. Metal-Organic Frameworks (MOFs) have recently emerged as an alternative to overcome these issues. Here, we propose a robust Zr-phosphonate MOF (UPG-1) bearing labile protons able to act *a priori* as an efficient electrolyte in PEMFCs. Further, in an attempt to further enhance the stability and conductivity of UPG-1, a proton carrier (the amino acid Lysine, Lys) was successfully encapsulated within its porosity. The behaviors of both solids as an electrolyte were investigated by a complete experimental (impedance spectroscopy, water sorption) and computational approach (MonteCarlo, water sorption). Compared with the pristine UPG-1, the newly prepared Lys@UPG-1 composite showed similar proton conductivity but a higher stability, which allows a better cyclability. This improved cyclability is mainly related to the different hydrophobic-hydrophilic balance of the Lys@UPG-1 and UPG-1 and the steric protection of the reactive sites of the MOF by the Lys.

## 1. Introduction

Several innovative technologies have been discovered and used for the green production of energy avoiding pollutant emissions to atmosphere and, consequently, environmental damages (e.g., global warming, water and air contamination, among others). Fuel cells (FCs), in particular proton-exchange membrane fuel cells (PEMFCs), have attracted a great worldwide scientific interest due to the possibility to generate electrical power by using carbon-free fuels (e.g., H_2_) [1,2,3]. Nowadays, those devices are the most promising clean energy systems for electric vehicles (in terms of specific energy are 5 times higher than rechargeable batteries) due to their capacity to make engines work, while consuming the environmentally friendly hydrogen and producing only water as sub-product [4]. Although relevant advances have been achieved toward the development of PEMFC components (i.e., electrodes, anode and cathode and electrolyte), the majority of the efforts faces the challenge of developing efficient and durable electrolyte materials, responsible for the conduction of protons from the anode to the cathode [5,6,7,8,9]. The main problem that the current electrolytes present is the necessity of a complex active humidification process associated with degradation and, consequently, loss of efficiency [10]. In this sense, besides a high proton conductivity (> 10^−2^ S·cm^−1^), an ideal electrolyte must possess other requirements for insuring the successful proton transfer reaction, such as—(i) possibility of molecular reorientation; (ii) presence of a flexible hydrogen-bond network; (iii) a driving force, being usually a concentration gradient; (iv) good thermal and chemical stability; (v) chemical compatibility with other cell components (for instance, electrodes); (vi) membrane shaping; (vii) low cost; and (viii) ease of production in large amounts.

Among a huge variety of solid materials, including organic polymers (e.g., the most used Nafion^®^) [11]), zeolites [12], perovskites [13], polyoxometalates [14] and covalent organic frameworks (COFs) [15], metal-organic frameworks (MOFs) have emerged as promising materials for the preparation of proton-conducting electrolytes [8,10,16,17,18,19,20]. Their chemical, thermal and mechanical stability in symbiosis with their intrinsic structural properties (e.g., easy tunable composition and topology and high regular porosity-up to S_BET_ = 7000 m^2^·g^−1^ [21,22], allowing the hosting of chemical species) offer the chance to create defined and efficient conductive pathways toward high proton conductivities. Thus, two main strategies have been reported to prepare high proton-conducting MOFs—(i) an intrinsic conductivity coming from the hybrid framework by using phosphonate-, sulfonate- or carboxylate-based coordinating ligands with or without additional free functional groups bearing acidic protons, responsible to tune the p*K*a values (R = -NH_2_, -SO_3_H, -OH, -CO_2_H) [23,24,25,26]; and (ii) insertion of proton carriers into the porous network such as counterions and/or neutral acids and dyes (e.g., NH_4_^+^, H_2_SO_4_, H_3_PO_4_, imidazole, 1*H*-1,2,4-triazole and adipic acid, among others) [27,28,29,30,31,32,33]. 

Based on the literature, the second strategy, that is, incorporation of proton-carrier guests, has been employed for the preparation of two types of MOFs—anhydrous and water-mediated proton-conductive MOFs [10,17,34]. Furthermore, such chemical species play a crucial role on their hydrophilic/hydrophobic character toward water adsorption, being essential for water-mediated proton-conductivity. In this line, Khatua et al. reported the synthesis of a Cu^I^-based MOF (formulated as [C_44_H_48_N_6_O_9_Cu_2_I_2_]*_n_*) with a very low proton conductivity [31], which was impressively improved (ca. 7 orders of magnitude from 10^−10^ to 10^−3^ S·cm^−1^) by tuning the water adsorption through the insertion of a series of guest molecules into the MOF’s porosity (e.g., dimethylformamide, diethylamine, nitrobenzene, 1,4-dinitrobenzene, pyridine and 1*H*-1,2,4-triazole). Recently, Liu and co-workers also assessed the influence of the encapsulation of a dye (8-hydroxy-1,3,6-pyrenetrisulfonic acid trisodium salt) into the microporous cationic indium carboxylate FJU-10, on its proton conductivity [29]. Both FJU-10 and dye@FJU-10 confine abundant water into their channels, favoring proton conduction. Further, the introduction of the dye 8-hydroxy-1,3,6-pyrenetrisulfonic acid trisodium salt helps the formation of hydrogen-bonds between the encapsulated species and water molecules, facilitating the proton transport and, consequently, enhancing 5-fold the proton conductivity (from 1.57 × 10^−3^ to 7.50 × 10^−3^ S·cm^−1^ at 90 °C under no additional humidity). As a last example, Ponomareva et al. have used the well-known mesoporous chromium(III)-terephthalate MIL-101 as a solid platform to encapsulate strong acids (i.e., CF_3_SO_3_H, *p*-CH_3_C_6_H_4_SO_3_H, H_2_SO_4_ and H_3_PO_4_) in order to prepare high proton-conducting acid@MIL-101 solids with robust structures [33,35]. For all the cases, the proton conductivity increases according to the concentration and nature of the acids as well as with the humidity. Conductivity values of CF_3_SO_3_H@MIL-101 and H_2_SO_4_@MIL-101 solids (8.0 and 6.0 × 10^−2^ S·cm^−1^, respectively) are comparable with the best reported proton conductors, such as Nafion^®^.

**Figure 1:**
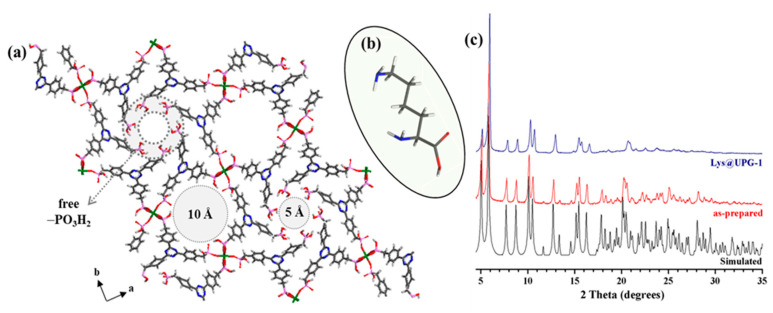
(**a**) Crystal structure of UPG-1 viewed along the *c* axis (crystallization water molecules are omitted for the sake of clarity), exhibiting the presence of the 5 and 10 Å channels and uncoordinated phosphonic acid groups (-PO_3_H_2_). (**b**) Structural representation of lysine. (**c**) Powder X ray diffraction (PXRD) patterns of simulated and as-prepared UPG-1 and Lys@UPG-1. Color code for UPG-1 and lysine: C = dark grey, H = light grey, N = blue, O = red, P = rose and Zr = green.

Following this promising strategy, the microporous Zr phosphonate MOF UPG-1 (Zr[H_4_ttbmp]_2_·10H_2_O; H_6_ttbmp = 2,4,6-tris(4-(phosphonomethyl)phenyl)-1,3,5-triazine; Figure 1a) [36] was selected here as promising original proton conductor MOF due to some interesting structural features—(i) the structure is based on 1D chains of ZrO_6_ octahedra connected by PO_3_C tetrahedra from the linker, additionally exhibiting one uncoordinated -PO_3_H_2_ group and two coordinated -PO_3_H groups per ligand, which leads to eight uncoordinated –OH moieties per unit formula suitable to contribute to the proton conductivity by establishing H-bonding pathways; (ii) high porosity (i.e., 1D channels with diameters of 5 and 10 Å), well-adapted to host protons, donor/acceptor molecules (e.g., water) or complexes; (iii) high thermal, chemical and mechanical stability to be used at non-ambient conditions (i.e., high temperatures and relative humidities, RH); and, in addition, (iv) environmentally compatible chemical composition (i.e., abundant non-toxic Zr^4+^ cations and organic linker). Despite fulfilling the prerequisites for a good proton conductor, UPG-1 has been never proposed in this applicative field. Consequently, we here originally propose the evaluation of the proton conductive properties of UPG-1 as a function of temperature and RH, fully characterizing the solid upon the measurement conditions with the help of different techniques (complex impedance spectroscopy (CIS), powder X-ray diffraction (PXRD), Fourier transformed Infrared spectroscopy (FTIR), thermogravimetric analysis (TGA), elemental analysis, induced coupled plasma atomic emission spectroscopy (ICP-OES), nitrogen and water sorption isotherms and molecular simulations). Further, in an attempt to further improve its conductive performances and cyclability, the amino acid lysine (Lys; Figure 1b) was then inserted into the channels of UPG-1. Lys is a low cost, safe naturally-occurring and environmentally friendly amino acid rich in H^+^ donor/acceptor sites due to the presence of two –NH_2_ and one –CO_2_H functional groups. These moieties are able to interact, through the formation of H-bonding contacts, with both UPG-1 and adsorbed water molecules as well as Lys-Lys molecules, leading to the formation of proton-conducting pathways. To the best of our knowledge, this is the first time that an amino acid is encapsulated in a MOF to play the role of a hosted proton carrier. Only quite few proton-conducting amino acid-based MOFs have been reported with such bio-organic molecules, being used as primary building units [37,38,39]. Finally, water adsorption capacity of UPG-1 and Lys@UPG-1 was investigated using a combination of experimental and simulation approaches (Grand Canonical Monte Carlo—GCMC), assessing the hydrophobic/hydrophilic character of both solids in order to understand their associated proton conduction and to identify plausible proton-conducting pathways.

## 2. Results

### 2.1. Synthesis and Characterization of UPG-1 and Lys@UPG-1

**Figure 2:**
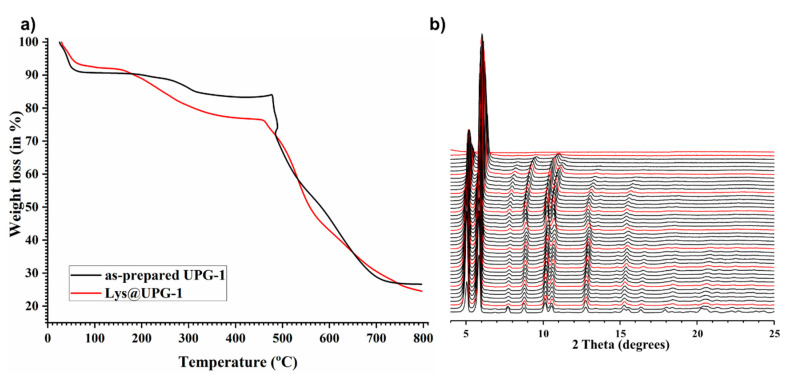
(**a**) Thermogravimetric analysis (TGA) of UPG-1 and Lys@UPG-1 and (**b**) VTPXRD patterns of UPG-1 from 30 to 500 °C. Each red line corresponds to an increment of 50 °C. No significant structural changes are observed up to ca. 440 °C, besides some shift of the peaks, at higher temperatures (i.e., from ca. 350 °C) and loss of crystallinity after ca. 400 °C. An amorphous residue appears at about 500 °C.

UPG-1 was synthesized according to a previously reported procedure [36], by reacting Zr^4+^ with H_6_ttbmp under soft conditions (80 °C for 48 h; see experimental section for further details). After reaction, UPG-1 structure was isolated sharing the same PXRD pattern profile than that simulated (Figure 1c). Its high thermal stability (up to ca. 440 °C) was also evidenced by TGA and variable-temperature powder X-ray diffraction (VTPXRD) studies (Figure 2), in agreement with the reported data [36].

Lys was successfully inserted into the MOF porosity (denoted as Lys@UPG-1) by using a simple diffusion/impregnation methodology. Briefly, after dehydration (150 °C for 5 h under primary vacuum), UPG-1 was suspended in a ethanolic Lys solution (UPG-1:Lys molar ratio of 1:20) and then, magnetically stirred at ambient temperature for 18 h (see Experimental Section for further details). Under these conditions, Lys@UPG-1 exhibits similar PXRD patterns than the pristine UPG-1 (Figure 1c), supporting that the diffusion of Lys into the 1D channels of the MOF does not induce significant structural changes in the MOF with the absence of free recrystallized Lys. In contrast with the pristine UPG-1, the composite material exhibits a rather low accessible porosity to N_2_ (Figure S1, *S*_BET_ = 15 m^2^·g^−1^; see discussion section).

Lys loading was quantified by TGA and elemental analysis. Both TGA curves (Figure 2) depict three main weight losses, being attributed to (1) the release of solvent molecules from the materials’ porosity (i.e., water for UPG-1 and, mostly, ethanol for Lys@UPG-1); (2) the formation of pyrophosphonates (quite frequent in phosphonate-based MOFs) [40], leading to a new dehydration of MOF structures and the release of Lys molecules for Lys@UPG-1; and (3) oxidation of the organic linker and the consequent collapse of the hybrid framework. Also, the thermal stability of the composite is comparable with that of the pristine UPG-1. Nevertheless, some important differences in the TGA curve profiles are observed. While the first weight loss is higher for UPG-1 than for Lys@UPG-1 (i.e., 10% vs. 7.9%, respectively), corresponding to a superior solvent adsorption capacity, the second one is much higher for the composite than for the pristine MOF (i.e., 4.7% vs. 14.9%, respectively). The latter is attributed to a Lys loading of ca. 10.2 wt.%, which is released in the 140–405 °C temperature range. Elemental analyses are in good agreement with the TGA [exp (%): C 44.43; H 4.31; N 8.90; P 12.19; Zr 5.84; theo (%): C 44.66; H 4.30; N 7.72; P 12.79; Zr 6.28; corresponding to a Lys content of ca. 10.1 wt.%; see Experimental Section], estimating thus one encapsulated Lys molecule per UPG-1 unit formula: [Zr(H_4_ttbmp)_2_]·0.9Lys·2H_2_O (corresponding to ca. 8 Lys molecules per unit cell-u.c.). 

With the aim to estimate the maximum loading of Lys in the UPG-1, molecular simulations were performed on the Lys-loaded UPG-1. First, preliminary calculations were carried out by employing force fields based on geometrical optimization of the fully dehydrated UPG-1, as well as density functional theory (DFT) geometry optimization for Lys molecule, to start the simulations on a robust solid structure, in order to calculate the partial charges required in GCMC studies following the computational procedure described in the Experimental Section (see Figures S2 and S3 in the ESI). Then, the theoretical pore size distribution of UPG-1 was calculated (Figure S4), obtaining pore sizes of 6 and 10 Å, in very good agreement with the crystal data (i.e., 5 and 10 Å). Finally, it was possible to determine the maximum amount of Lys able to be adsorbed in UPG-1 using GCMC calculations. Thus, the theoretical saturation of Lys was higher than experimental one (11 vs. 8 Lys per u.c.). Such deviation between experimental and theoretical approaches could be explained by the fact that these calculations for Lys saturation did not take into account the presence of the solvent from the encapsulation process, which could be co-adsorbed. As experimentally, theoretical Lys saturation was not obtained, empirical Lys content was considered for further calculations in order to assess the Lys conformation within the pores. In this line, the 3D density plot of Lys presence (Figure S5) illustrates that the Lys molecules are mainly adsorbed within the largest pores. The more energetic sites of the material are both -OH groups and O bonded to phosphate linkers, interacting with the -NH_2_ groups from Lys (see Figure S6). Secondary benzene rings can also be observed as interacting with the alkyl chains of the Lys molecules. In agreement with these observations, FTIR spectra of UPG-1 before and after Lys encapsulation (Figure S7) show band shifts consistent with the formation of weak interactions between the Lys and the MOF. In general, P-OH stretching band and skeletal vibrational bands of the benzene rings of the UPG-1 are slightly shifted to lower wavenumber (from 1010 to 990 cm^−1^ and from 1515 to 1511 cm^−1^, respectively), suggesting the interaction of the Lys with the phosphonate groups and benzene rings of the network. However, no bands corresponding to the Lys appear in the composite spectrum, probably due to the relatively low Lys content.

### 2.2. Evaluation of UPG-1 and Lys@UPG-1 Proton Conductivity

Despite the promising properties (namely thermal and chemical stability, the presence of labile protons and water molecules in the porous structure) of the UPG-1 to act as proton conductor, the proton conductivity of this material has not been tested until now. Thus, crystalline powdered UPG-1 and Lys@UPG-1 materials were first compacted (using uniaxial and isostatic pressure) as a 6 mm-diameter cylindrical-like disk. After confirming their structural stability upon shaping (Figure S8), their proton conductivity was assessed by CIS (at 70 and 90% RHs in the 30–90 °C temperature range; see Experimental Section for further details) using sputtered gold ion-blocking electrodes.

Typical impedance datasets are displayed in Figure S9. At high frequency, the data show an almost undistorted semicircle with an associated capacitance of 6.0 pF·cm^−1^ and is, therefore, attributed to the bulk resistance of the sample. In the low frequency region, it seems to appear an inclined spike with an associated capacitance of 4.3 µF·cm^−1^, which indicates a partial-blocking electrode response consistent with proton migration. The frequency dependent conductivity comprises at least two regions—the plateau at low frequencies and the dispersion at high frequencies. The plateau corresponds to constant conductivity coincident with the overall conductivity of the sample, as determined by the interception of the semicircle with the abscissa at the low frequency from the semicircles in the Nyquist plots. This behavior was found for both UPG-1 and Lys-UPG1. 

**Figure 3:**
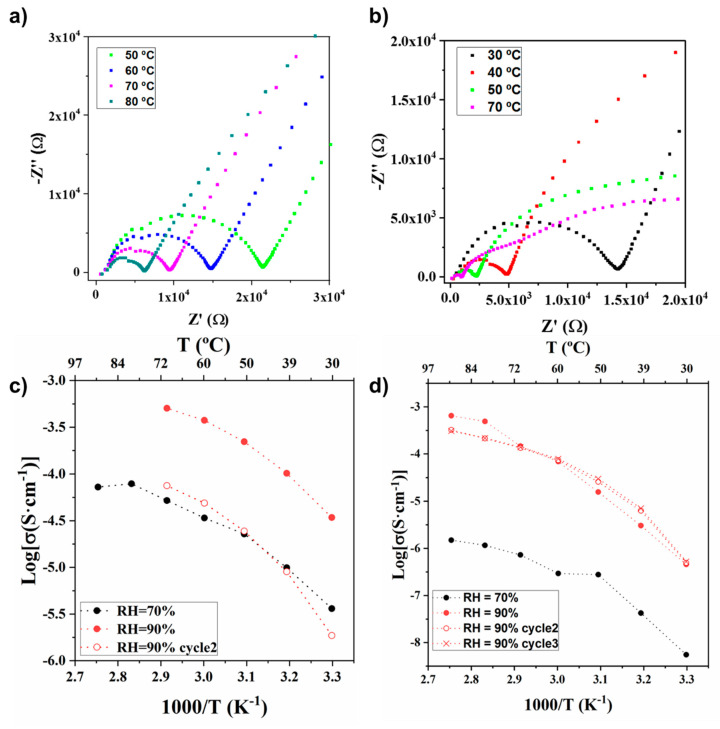
Nyquist plots for UPG-1 collected at 70% RH (**a**) and at 90% RH (**b**) and different temperatures. Arrhenius plots for UPG-1 (**c**) and Lys@UPG-1 (**d**), respectively, exhibiting the evolution of the proton conductivity vs. temperature at 70 and 90% RH. Recyclability of both UPG-1 and Lys@UPG-1 at 90% RH is also represented.

As expected, we can observe that the conductivity of UPG-1 and Lys@UPG-1 is strongly dependent on the temperature and humidity (Figure 3). In particular, proton conductivity increases by around one and two orders of magnitude when passing from 70 to 90% RH at 70 °C for UPG-1 (from 5.2 × 10^−5^ to 5.1 × 10^−4^ S·cm^−1^) and Lys@UPG-1 (from 7.2 × 10^−7^ to 1.5 × 10^−4^ S·cm^−1^) (Figure S10), respectively. However, both solids exhibit similar proton conductivities (around 10^−4^ S·cm^−1^) at high temperature, indicating the poor impact of the Lys encapsulation on the final conductivity values. Note here that these values are in the range of the reported ones for other phosphonate-based MOFs (Table S1), including the recently reported UPG-2 (10^−4^ S·cm^−1^) [41] which is based on an isomeric H_6_ttbmp linker and presents an identical chemical formula than UPG-1.

In order to understand the proton conduction mechanism, we analyzed the impedance values at different temperatures, while keeping the RH constant at 70% and 90% (Figure 3 and Figure S11). For the UPG-1 at 70% RH (Figure 3a), the Nyquist plots depict a single semicircle in all the temperature range; however for 90% RH, the plots change drastically at temperatures equal and higher than 40 °C presenting two or three semicircles (i.e., a well-defined semicircle at higher frequencies and a depressed semicircle at intermediate frequencies, being overlapped for a new one at low frequencies, Figure 3b). The occurrence of more than one semicircle in the impedance spectra of MOFs has been previously described, since the semicircle at high frequency corresponds to the bulk conductivity and the one at intermediate frequency is attributed to the intergrain resistance [26] or interparticle contact impedance in analogy to the grain boundaries’ resistance of the sintered electroceramic samples. Lys@UPG-1 sample shows a similar behavior (Figure 3d and Figure S11), although the conductivity values are slightly lower. This fact could be due to the location of the Lys molecules within the pores (Figure S12), restricting the movement of protons by steric hindrance. Considering this information, only the values of the high frequency semicircles, corresponding to the bulk conductivity, were taken into account to estimate the proton conductivity values. In Figure 3c,d, the evolution of the conductivity with the temperature for UPG-1 and Lys-UPG-1 is displayed. It is clear from the plots that the temperature dependent conductivity did not follow the Arrhenius behavior. Pseudo-activation energies (Ea) were calculated by fitting the conductivity data to the Vogel−Tamman−Fulcher (VTF) equation (Figure S13) [42], which is widely used for non-Arrhenius polymeric ion conductors. The non-Arrhenius behavior has been already described for other MOFs, although is not fully understood [43,44].

Table S2 summarizes the values for the three VTF parameters as obtained for our samples. The values of the pseudo-activation energy are within the range typically attributed to a Grotthuss transfer mechanism via water molecules (<0.5 eV) [45], which involves proton hops from a proton donor to an acceptor along a hydrogen bond (H-bond) and a reorientation where an H-bond is cleaved and the proton reorients to another proton acceptor [43,46]. The higher Ea in the composite could be associated to the restriction of the water molecules diffusion in micropores due to the presence of Lys when compared with the pristine UPG-1 without the amino acid and also with the lower amount of water molecules in the composite material when compared with the pristine material (2 vs. 10 per unit formula, respectively).

Finally, in order to assess the stability of the UPG-1 and Lys@UPG-1 samples under different temperature cycles, several recyclability measurement cycles at 90% RH were carried out (Figure 3d, Figures S14 and S15). As expected, impedance spectra exhibit same shapes (i.e., presence of three conductivity contributions) than the ones collected on the first cycle of experiments. In the case of the UPG-1, a drastic decrease in the conductivity of around one order of magnitude was noticed upon the second cycle at 70 °C and RH 90% (from 5.1 × 10^−4^ to 7.5 × 10^−5^ S·cm^−1^), which might be related with structural changes, as evidenced by the slight modification in the PXRD pattern after the second cycle (Figure S16). In contrast, the conductivity of the Lys@UPG-1 composite remains remarkably almost unaltered during the 2nd and 3rd cycles at 90% RH and 70 °C (Figure 3d and Figure S17). Further, Lys@UPG-1 showed no significant structural changes after the recycling process even at higher temperatures (90% RH and 90 °C) and additional cycles (from 6.5 × 10^−4^ and 3.1 × 10^−4^ S·cm^−1^ after 3 cycles (Figure 3d and Figure S17).

### 2.3. Water Adsorption Properties of UPG-1 and Lys@UPG-1

**Figure 4:**
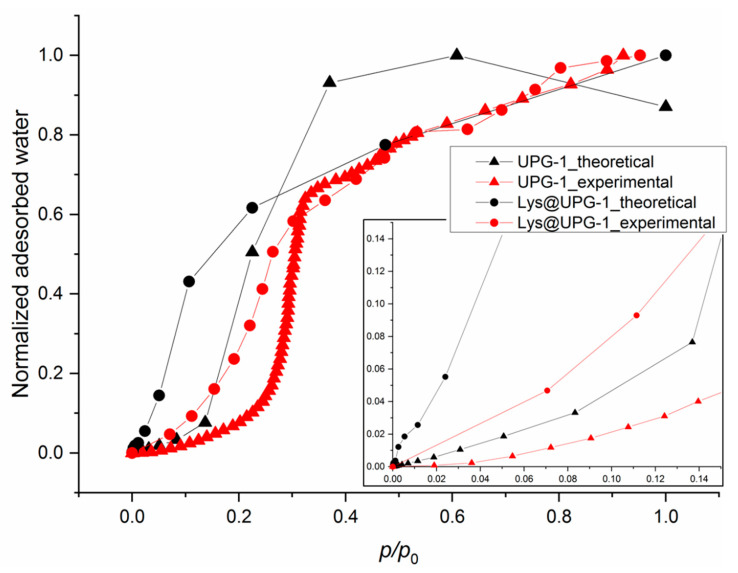
Normalized water adsorption isotherms of UPG-1 and Lys@UPG-1 obtained by molecular simulations (at 25 °C) and experimentally (at 20 °C).

In order to shed some light on the main parameters involved in the mechanism of proton conduction, water sorption capacities of both UPG-1 and Lys@UPG-1 (and thus, their hydrophilic/hydrophobic character) were estimated by using GCMC simulations and validated by experimental sorption measurements. For the pristine UPG-1, a theoretical adsorption enthalpy value for water of ca. −23.8 kJ·mol^−1^ was thus obtained by determining the interaction of the first adsorbed water molecule with the MOF (see Figures S18 and S19 (left)). The water adsorption isotherm of the fully dehydrated UPG-1 was modelled up to the saturation at 300K. Although the theoretical isotherm presents a maximum water adsorption of ca. 150 mg·g^−1^ at *p*/*p*° = 1, in the experimental one the maximum adsorption is ca. 98 mg·g^−1^ at *p*/*p*° = 1 (Figure 4 and Figure S20). This difference can be explained by an incomplete activation of the solid, by some structural modifications (rotation of some parts of the structure) upon water adsorption or by difficulty to precisely reproduce the behavior of hydrophobic solids using classical force fields [47] (even if the theoretical saturation is generally only related to the free volume). Nevertheless, the same sigmoid-like shape is observed. 

On the other hand, configuration of the UPG-1 containing 8 Lys per u.c. (see above) was the starting point of GCMC calculations for the water adsorption in Lys@UPG-1. The theoretical adsorption enthalpy for low water coverage for the composite (−70 kJ·mol^−1^) is higher than the liquefaction enthalpy (equal to −41 kJ·mol^−1^). Both sorption isotherms, theoretical and experimental, for the Lys@UPG-1 present the same amount of adsorbed water at saturation (ca. 100 mg·g^−1^ at *p*/*p*° = 1; Figure 4). However, there is a noticeable difference in the adsorption curves between the theoretical and the experimental sorption isotherms. It can be observed that the water adsorption is shifted at higher relative pressure for the experimental isotherms, similarly to the UPG-1. However the experimental evolution of the adsorption isotherms for UPG-1 and Lys@UPG-1 is well reproduced by molecular simulations, which allows us to discuss the main adsorption mechanisms occurring in the UPG-1 pores. 

## 3. Discussion

In the present study, the amino acid Lys was successfully encapsulated within the UPG-1 porosity with the aim to improve the *a priori* proton conductor behavior of UPG-1. The resulting Lys@UPG-1 composite, containing ca. 8 Lys molecules per u.c., not only keeps intact its crystalline structure but also exhibits a permanent porosity accessible to N_2_ (Figure S2, *S*_BET_ = 15 m^2^·g^−1^), which is not the case for the pristine UPG-1. Despite the presence of 5 and 10 Å channels in the structure of the pristine UPG-1 and the previously confirmed accessible porosity to other gases (CO_2_, CH_4_ and C_4_H_10_), its microporosity is not accessible to N_2_ [36]. This fact was explained by the low affinity (at 77, 273 and 298 K) and slow diffusion kinetics of N_2_ (at 77 K) [36]. Thus, the presence of the Lys in the network might slightly modify the conformation of the structure (according to the FTIR data; Figure S7), making the N_2_ diffusion inside the channels easier. 

Concerning the performances of both solids as electrolyte, the measurements of proton conductivity show a significant increase with the RH, as expected. At 70% RH, the Nyquist plots show a single semicircle, attributed to the bulk resistance of the solid. On the other hand, at 90% RH, the plots change drastically at temperatures equal and higher than 40 °C, presenting a well-defined semicircle at higher frequencies and a depressed semicircle at intermediate frequencies, being overlapped for a new one at low frequencies (Figure 3b). This new contribution, with respect to lower RH, could be associated to an extrinsic transport through interparticle phases described by Tominaka and Cheetham for MOFs [48]. These observations suggest that the proton conductivity of UPG-1 and Lys@UPG-1 might depend not only on the protons belonging to frameworks and/or in the micropores (intrinsic) but also on the hydrated interparticle phases (extrinsic conductivity). On the other hand, the cyclability test results affirm the robustness of the Lys@UPG-1, a key feature for potential applications as a proton-conducting material.

Therefore, in order to rationalize the obtained proton conductive behavior, we evaluate here the role of the water interaction with the structure (i.e., hydrophobic/hydrophilic balance) by experimental and simulated water sorption isotherms. First, it is known that molecular simulation is a useful way to unveil the hydrophilic/hydrophobic character of solids through the determination of—(i) adsorption enthalpies for water, since values lower than ca. −41 kJ·mol^−1^, that is, inferior to the liquefaction enthalpy for water, are characteristics to hydrophobic compounds; (ii) the shape of water adsorption isotherms; and (iii) the comparison of interactions between water molecules and solid’s frameworks and water-water contacts in order to assess if water prefers to interact with the solid (characteristic for compounds with hydrophilic character) or with other water molecules (typical of compounds with hydrophobic character) [49]. In addition, simulation data have been validated here by experimental sorption isotherms (see below). Thus, the poor water adsorption at low pressures (*p*/*p*° < 0.2) and the reduced value of adsorption enthalpy estimated by GCMC calculations (ca. −23.8 kJ·mol^−1^) confirm the hydrophobic character of UPG-1 (Figure 4). It was therefore possible to study the existing interactions between water molecules and the framework and/or water-water molecules from the simulations. Indeed, the determination of the main adsorption sites of the UPG-1 was possible and the water molecules were localized close to the -OH groups bonded to the phosphate groups (see Figures S18, S19 (left) and S21). The corresponding interaction distances observed from GCMC calculations showed that the interactions between water molecules and those between water molecules and UPG-1 framework were relatively equivalent (between 1.8–2.0 Å). This behavior was similar to that elucidated in MIL-53 and confirms the hydrophobic character of the UPG-1 [47]. It follows that the proton conduction ways were mainly based on the H-bonds existing between the water molecules present in the pores, the framework being not strongly involved in the conduction process. An illustration of the net strong interactions between water molecules was given in Figure S21.

Remarkably, the behavior of the Lys@UPG-1 was clearly different from the pristine UPG-1 (see Figure 4). The adsorption of water at lower pressures (*p*/*p*° < 0.08) with a calculated enthalpy ca. −70.0 kJ·mol^−1^ (higher than the water liquefaction enthalpy) indicates a hydrophilic character of the composite. Further, the 3D density plots of water presence within the composite (see Figures S19 (right) and S24) suggests that water molecules interact with the Lys in both pores. The investigation of the snapshots obtained from GCMC calculations illustrated that similar proton conductive ways were present in the pores but here the Lys molecules participate also in the conduction mechanisms (see distances between water molecules and Lys in Figure S24). In contrast with the UPG-1, where the distribution of the water molecules imposes isotropic conductive ways, the presence of Lys within the pores might limit the organization of the water molecules, leading to more directive conduction ways. However, the saturation of the 1D channels of the MOF with Lys molecules may also limit the entrance of high amounts of water, necessary for the formation of efficient proton-conducting pathways. Thus, in contrast to the expected, the introduction of Lys does not apparently promote an improvement on the proton conductivity of UPG-1.

Finally, UPG-1 exhibits an important decrease in its proton conductivity after recycling, associated to some modification/degradation of its framework (e.g., defects; see PXRD data Figure S16). In contrast, it is interesting to note that modifying the hydrophilic/hydrophobic balance of the Lys@UPG-1, the presence of Lys significantly improves both the thermal and aqueous stability of the composite, leading to a higher cyclability (3 cycles). The higher stability could be explained by the protection of the UPG-1 reactive sites by steric hindrance as a consequence of the presence of Lys adsorbed on its surface [50], making that the water interacts preferentially with the -NH_2_ groups of the Lys instead the -OH groups from the material (see Figures S19 and S24). Considering the poor stability of the current electrolyte materials (mainly Nafion^®^) [10,11], this observation is of high relevance for the future preparation of PEMFC devices based on robust solids, among other applications (e.g., as solid state electrolyte for metal-ion rechargeable batteries).

## 4. Materials and Methods 

### 4.1. Reagents and Solvents

Chemicals were readily available from commercial sources and used as received without further purification—zirconium(IV) oxychloride octahydrate (ZrOCl_2_·8H_2_O, 98%, Sigma Aldrich, Madrid, Spain); lysine (C_6_H_14_N_2_O_2_, 98%, Sigma Aldrich); hydrofluoric acid (HF, 49%, J.T. Baker, Madrid, Spain); methanol (MeOH, p.a., Chem-Lab, Madrid, Spain); ethanol (EtOH, p.a., Chem-Lab).

### 4.2. Synthesis of [Zr(H_4_ttbmp)_2_]·10H_2_O (UPG-1)

UPG-1, as well as its corresponding organic linker (i.e., 2,4,6-tris[4-(phosphonomethyl)phenyl]-1,3,5-triazine; H_6_ttbmp), were synthesized according to the procedure reported by Taddei et al. with slight modifications [36]. ZrOCl_2_·8H_2_O (0.2852 g, 0.887 mmol) was dissolved in 16.1 mL of a HF solution (2.9 M) in a 103 mL Teflon-lined autoclave reactor. Then, to this solution, 11.5 mL of methanol, 23 mL of distilled water and 0.5428 g of H_6_ttbmp (0.918 mmol) were added. The reactive mixture was stirred at ambient temperature for 5 min and then, transferred to an oven where the reaction took place at 80 °C for 48 h. The resulting white powder was washed with distilled water, a distilled water/methanol (3:1) mixture and methanol and dried at ambient temperature.

Elemental analyses for UPG-1 (i.e., considering the dehydrated form of UPG-1, [Zr(H_4_ttbmp)_2_], after activation at 100 °C for approximately 24 h). Theoretical (wt.%): C 45.40; H 3.49; N 6.62; P 14.6; Zr 7.18. Found (wt.%): C 45.05; H 3.59; N 7.12; P 13.7; Zr 6.74.

TGA data (weight losses in %) and derivative thermogravimetric peaks (DTG; in italics inside the parentheses) for UPG-1: 23–100 °C −10% (45 °C); 150–400 °C −4.7% (283 °C).

### 4.3. Preparation of Lys@UPG-1

UPG-1 (0.250 g, 0.17 mmol) was added to a sealed three-necked round-bottom flask and outgassed at 150 °C for 5 h under primary vacuum. A solution composed of Lys (0.497 g, 3.40 mmol) in 75 mL of ethanol was added and the encapsulation procedure occurred at ambient temperature during 18 h under constant magnetic stirring. The desired solid compound Lys@UPG-1 was recovered by centrifugation (13,000× *g* rpm for 7 min) and washed twice with ethanol. After each washing step, Lys@UPG-1 was recovered by centrifugation (13,000× *g* rpm for 7 min).

Elemental analyses for Lys@UPG-1 *(*i.e., considering the empirical formula [Zr(H_4_ttbmp)_2_]·Lys·2H_2_O, after activation at 100 °C for 24 h). Theoretical (wt.%): C 44.66; H 4.30; N 7.72; P 12.79; Zr 6.28. Found (wt.%): C 44.43; H 4.31; N 8.90; P 12.19; Zr 5.84.

TGA data (weight losses in %) and derivative thermogravimetric peaks (DTG; in italics inside the parentheses) for Lys@UPG-1: 29–125 °C −7.9% (45 °C); 140–405 °C −14.9% (234 °C).

### 4.4. General Instrumentation

PXRD patterns were collected from 4 to 35° (2θ) using a step size of 0.02° and 2.5 s per step in continuous mode in an Empyream PANALYTICAL diffractometer, equipped with a PIXcel3D detector and with a copper radiation source (Cu Kα, λ = 1.5406 Å), operating at 45 kV and 40 mA. Nitrogen sorption isotherms were collected at 77 K using a Quantachrome Autosorb. Prior to the analysis, the samples were activated at 100 °C under primary vacuum for 16 h. TGA were carried out using a SDT Q-600 thermobalance (TA instruments) under air flow (100 mL·min^−1^) with a heating rate of 5 °C min^−1^ between room temperature and 800 °C. FTIR spectra were collected in the 4000 to 400 cm^−1^ range using a Thermo Nicolet 6700 FTIR with attenuated total reflectance (ATR) accessory instrument (Thermo scientific, ‎Waltham, MA, USA). Elemental analyses from light elements were carried out in a Flash 2000 analyzer from Thermo Scientific. Zr and P were measured by induced coupled plasma atomic emission spectroscopy (ICP-OES) using a 2300DV spectrometer from PerkinElmer. VTPXRD data for UPG-1 was collected on a D8 Advance Bruker AXS θ–2θ diffractometer (Cu Kα X-radiation, λ = 1.54060 Å), equipped with a LYNXEYE XE detector, operating at 40 kV and 40 mA and an Anton Paar XRK 900 high-temperature chamber. Intensity data for UPG-1 were collected in the step mode (0.03° 2θ, 1 s per step) in the range ca. 4 ≤ 2θ° ≤ 25. Data were collected between 30 and 800 °C in intervals of ranging from 10 to 50 °C. The heating ramp used was 5 °C·min^−1^.

### 4.5. Proton Conductivity

Proton conductivities (*σ*) of UPG-1 and Lys@UPG-1 were investigated by Complex impedance spectroscopy on compacted pellets of crystalline powders with diameters and thicknesses of 6.0 and 1.40 mm and 6.0 and 1.44 mm, respectively. Firstly, pellets were shaped by uniaxial pressure, applying 6.9 MPa for 5 min and then, isostatically at 4.9 and 19.6 MPa for 10 and 2 min, respectively. Their apparent densities were calculated, taking into account their weight and geometric dimensions (i.e., diameter and thickness). Both sides of the pellet were coated by Au ion-blocking electrodes by sputtering in a Leica EM ACE 200 instrument. The electrical measurements were performed on a parallel-plate capacitor configuration in air atmosphere. Measurements were carried out using an Impedance/Gain-Phase Analyzer SI 1260 (Solartron, UK), applying a 100 mV amplitude signal in the in the 10^−1^–10^7^ Hz frequency range. Measurements at different temperature (30–90 °C) and RHs of 70 and 90% were performed in a programmable climatic chamber (BINDER, UK). In order to ensure the reproducibility of all measurements, a dwell time of 15 min was defined for the system to reach stable conditions. By using this method, the RH and temperature could be controlled up to ±1% and ±1 °C, respectively. Impedance data analysis was performed using the ZView2 program [51]. Note here that conductivity measurements at 90%RH and 90 °C for UPG-1 have been omitted due to bad electrode contact.

The *σ* (in S·cm^-1^) was calculated by the following equation (Equation (1)):

(1) $$\sigma =\frac{l}{R\;X\;A},$$

where *l* and *A* are the thickness (cm) and the area (cm^2^) of the pellets, respectively. *R* is the ohmic resistance (Ohm) obtained from the intersection of the in Nyquist plot of the impedance curve with axis of the real component of the impedance.

The activation energy was estimation using the following equation (Equation (2)), employed for non-Arrhenius polymeric ion conductors

(2) $$\sigma =\sigma_{0}*exp\left(\frac{-E_{a}^{VKT})}{K\left(T-T_{0}\right)}\right),$$

where *σ*_0_ is the prefactor, *T* is the absolute temperature, *K* is the Boltzmann constant, *E_a_^VFT^* is the pseudoactivation energy and *T*_0_ is related in polymers to glass transition temperature at which the “free” volume disappears or at which configuration free entropy becomes zero. In this system also could be related to that temperature in which molecular water motions cease).

### 4.6. Water Adsorption Measurements

The water vapor sorption isotherms have been performed using a home-built device already described elsewhere [52]. This set-up is based on manometric measurements using capacitive pressure gauges (0–10 Torr and 0–1000 Torr). The sample cell can be unplugged from the sorption device to undergo a thermal treatment. Based on their thermal stability, the materials were activated at 150 °C for 8h under a vacuum of 10^−5^ Torr. The adsorption device sets initial pressures rather than equilibrium pressures allowing a more precise description of the sorption isotherms [53]. Water vapor sorption was performed at 313 K, which allows a large extent of water vapor to be produced (up to 57 Torr at saturation). A duration of 300 s at the same pressure in the sample cell was chosen as criterion for the thermodynamic equilibrium. Longer equilibration times gave the same sorption isotherms. Depending on the relative pressure and therefore the sorption process, different adsorption times could be observed.

Deionized water was used as sorbate. It was further put under low pressure in order to outgas dissolved oxygen.

### 4.7. Theoretical Calculations

In complement to the experimental part, a classical geometry optimization was performed with GULP code on the experimental structure of the dehydrated UPG-1 solid with all the H atoms initially placed at tabulated distances as a function of the bonded atoms. For that purpose, partial charges (Figure S2) for the solid were extracted using the qEq methodology based on electronegativity equalization approach and then combined with Universal Force Field (UFF) for Lennard Jones parameters [54]. The so-obtained structure was the starting point for Monte Carlo simulations to study the impact of water adsorption. In complement, for Lys molecule, DFT calculations were performed with GGA/PW91 functional (see Molecular simulation section in the ESI for more details), considering all electrons and using the DNP basis set proposed in DMol^3^. Convergence criteria are fixed at high sensibility for the integration accuracy, SCF tolerance and orbital cutoff quality. The ESP charges were thus extracted and reported in Figure S3.

The pore size distribution was determined using the Gelb and Gubbins methodology presented in the literature [55].

Calculations in the Grand Canonical ensemble were performed at 300 K to determine the adsorption isotherm for water (see Figure 4), the saturation loading in water molecules inside the UPG-1 structure and to extract the plausible configurations of water clusters inside the pores. 10 × 10^6^ steps for equilibration and 10 × 10^6^ steps of production were considered. In these calculations, the chemical potential of the adsorbate (here the water), the volume of the calculation cell and the simulated temperature are imposed. For that purpose, Ewald summation was also used for calculating the electrostatic interactions while the short-range contributions corresponding to Lennard Jones parameters were computed by applying the Lorentz-Berthelot rules applied on UFF force field parameters for UPG-1 atoms. The simulations were conducted using the previously simulated structures that were considered rigid, but with multicells (corresponding to 1 × 1 × 6 unit cells), in order to obtain cell parameters allowing us to impose a cut-off distance for Lennard Jones interactions equal to 12 Å. For water model, TIP4P-2005 was implemented for both electrostatic and Lennard-Jones parts [56].

The same procedure was then used for Lys@UPG-1, containing the same amount of Lys than experimental results. Again, UFF was considered for Lys atoms in the case of Lennard Jones parameters to reproduce the Van der Waals interactions between UPG-1 framework and the H_2_O molecules.

## 5. Conclusions

As a strategy to improve both the proton conductivity performance and stability of the Zr-phosphonate UPG-1, the amino acid lysine (Lys) has been encapsulated within its porosity as proton carrier using a simple and green impregnation method. With loadings of ca. 10 wt.% of Lys, both loaded-solids present conductivity values within the typical range of MOFs (around 5 × 10^−4^ S·cm^−1^).

The temperature dependence of proton bulk conductivity follows the VTF behavior, with a very low pseudo activation energy for both materials (Ea = 0.13 eV for Lys@UPG1 and 0.02 eV for UPG-1), indicating that the protons “hop” through the channels following the Grotthuss mechanism. The presence of an additional contribution in impedance plots of wet samples could be related to interparticle mobility, which is detectable for high temperature and RH. Further, the proton conductivity strongly depends on humidity, increasing by more than 1 and 2 orders of magnitude between 70% and 90% RH, suggesting that the water molecules present in the framework (including micropores) and/or in between particles are involved in the proton conduction. Although, unexpectedly, the insertion of a proton carrier does not lead to an increase of the proton conductivity, the Lys seems to play a protective role in the structure, keeping the material stable upon several cycles. This fact could be related to the higher hydrophilic character of the Lys@UPG-1 and to the Lys steric hindrance in the pores, protecting the MOF metallic centers.

## Supplementary Materials

The following are available online, Figure S1: N_2_ sorption isotherm at 77 K of Lys@UPG-1, Figure S2: Schematic representation of the partial charges calculated for the fully dehydrated UPG-1: (left) coordination geometry of Zr and (right) H_6_ttbmp organic linker, Figure S3: Schematic representation of the partial charges calculated for lysine, Figure S4: Pore size distribution of UPG-1 calculated by theoretical Monte Carlo simulations illustrating the presence of two type pores, corresponding to the channels with different diameter, Figure S5: 3D density plot for presence of lysine molecules in pristine UPG-1 calculated from Monte Carlo simulations, Figure S6 Snapshots depicting the interactions between the Lys molecules with the framework of Lys@UPG-1 obtained by GCMC simulations Figure S7: FTIR of Lys (blue) as-prepared UPG-1 (black) and Lys@UPG-1 (red), Figure S8: PXRD patterns of as-prepared UPG-1 (black) and UPG-1 (red) and Lys@UPG-1 (blue) after being pelletized at 19.6 MPa, Figure S9: Impedance data sheet of UPG-1, Figure S10: Impedance spectra from UPG-1 (a) and Lys@UPG-1 (b) at different relative humidity, Table S1: Proton conductivity values of different phosphonate MOFs, Figure S11: Nyquist plots for Lys@UPG-1 collected at 70% RH (a) and 90% RH (b) and different temperatures, Figure S12: Lys@UPG-1 structure along the c axis. Grey circles highlight the pores occupied by the Lys molecules, Figure S13: VTF fitting of the conductivity data from UPG-1 (top) and Lys@UPG-1 (bottom), Table S2: Values for the parameters of the VTF Eq. (Equation (2)) obtained by fitting the conductivity data for the two samples, Figure S14: Nyquist plots for UPG-1 collected at 90% RH and different temperatures (cycle 2), Figure S15: Nyquist plots for Lys@UPG-1 collected at 90% RH and different temperatures (cycle 2 (a) and cycle 3 (b)), Figure S16: PXRD of the pressed UPG-1 before (black) and after (red) proton conductivity measurements, Figure S17: PXRD of the pelletized Lys@UPG-1 before (black) and after (red) proton conductivity measurements, Figure S18: 3D density plot for the presence of water molecules (in red) in pristine UPG-1 calculated from Monte Carlo simulations in the two types of pores, Figure S19: Snapshots depicting the interactions between the first water molecule with the frameworks of (left): UPG-1 and (right): Lys@UPG-1 and Lys, obtained by GCMC simulations, Figure S20: Water adsorption isotherms of UPG-1 and Lys@UPG-1 obtained by molecular simulations (at 25 °C) and experimentally measured (at 20 °C), Figure S21: Snapshots depicting the interactions between the water molecules bonded with the frameworks of UPG-1 in the pores of 5 and 10 Å (a and b, respectively), obtained from GCMC simulations, Figure S22: Illustration of the main proton conductive ways involving the water molecules in pristine UPG-1, obtained from GCMC simulations. All the distances represented in pink are hydrogen bonds with lengths lower than 2.2 Å, Figure S23: 3D density plot for presence water in Lys-loaded UPG-1 calculated from Monte Carlo simulations, Figure S24: Illustration of the main proton conductive pathways involving both the water and Lys molecules in Lys@UPG-1, obtained from GCMC simulations. The distances reported in green (respectively in yellow) correspond to hydrogen between water molecules (respectively between water and Lys molecules).
