# Supplementary material for: Proton Conductive Zr-Phosphonate UPG-1—Aminoacid Insertion as Proton Carrier Stabilizer
Source: Molecules. 2020 Jul 31;25(15):3519. doi: 10.3390/molecules25153519 (PMC7436027; doi:10.3390/molecules25153519)
Supplement: Supplementary file 1 [file molecules-25-03519-s001.pdf]

Electronic Supporting Information

Proton conductive Zr-phosphonate UPG-1: aminoacid insertion as proton carrier stabilizer

Sérgio M. F. Vilela [1], Pablo Salcedo-Abraira [1], Alejandro Gómez-Peña [1], Philippe Trens[2], Alejandro Várez [3,*], Fabrice Salles [4,*], Patricia Horcajada [1,*]

[1] *Advanced Porous Materials Unit (APMU), IMDEA Energy, Avda. Ramón de la Sagra 3, E-28935 Móstoles, Madrid, Spain*

[2] *Institut Charles Gerhardt de Montpellier (ICGM) UMR 5253, Matériaux Avancés pour la Catalyse et la Santé, ENSCM/CNRS/UM, 240 Av. Prof. Jeanbrau, 34296 Montpellier Cedex 05, France*

[3] *Department of Materials Science and Engineering and Chemical Engineering, Universidad Carlos III de Madrid, Avda. Universidad 30, E-28911 Leganés, Madrid, Spain*

[4] *Institut Charles Gerhardt de Montpellier (ICGM) UMR 5253 CNRS UM2, Université de Montpellier 2, Place E. Bataillon, 34095 Montpellier Cedex 05, France*

* Corresponding authors e-mail: fabrice.salles@umontpellier.fr; alvar@ing.uc3m.es; patricia.horcajada@imdea.org

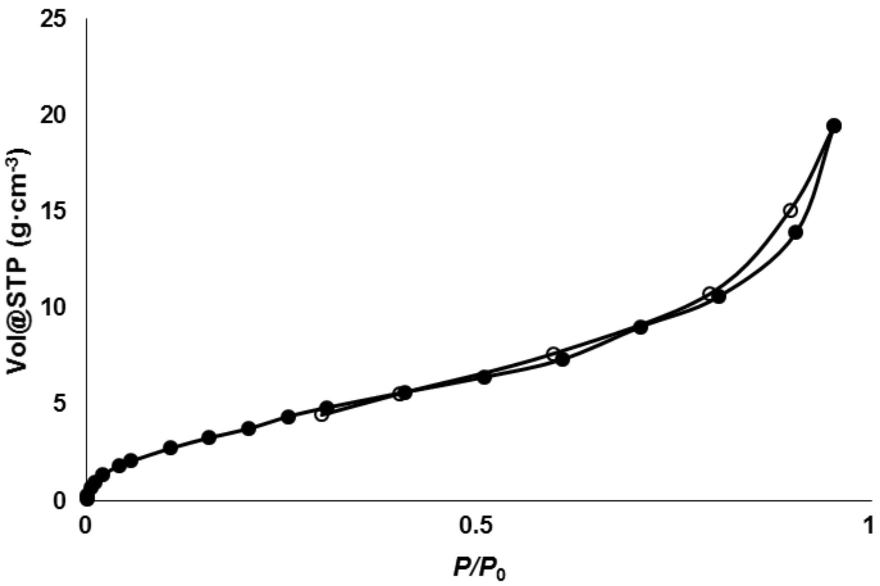

Fig. S1: N_2 sorption isotherm at 77K of Lys@UPG-1.

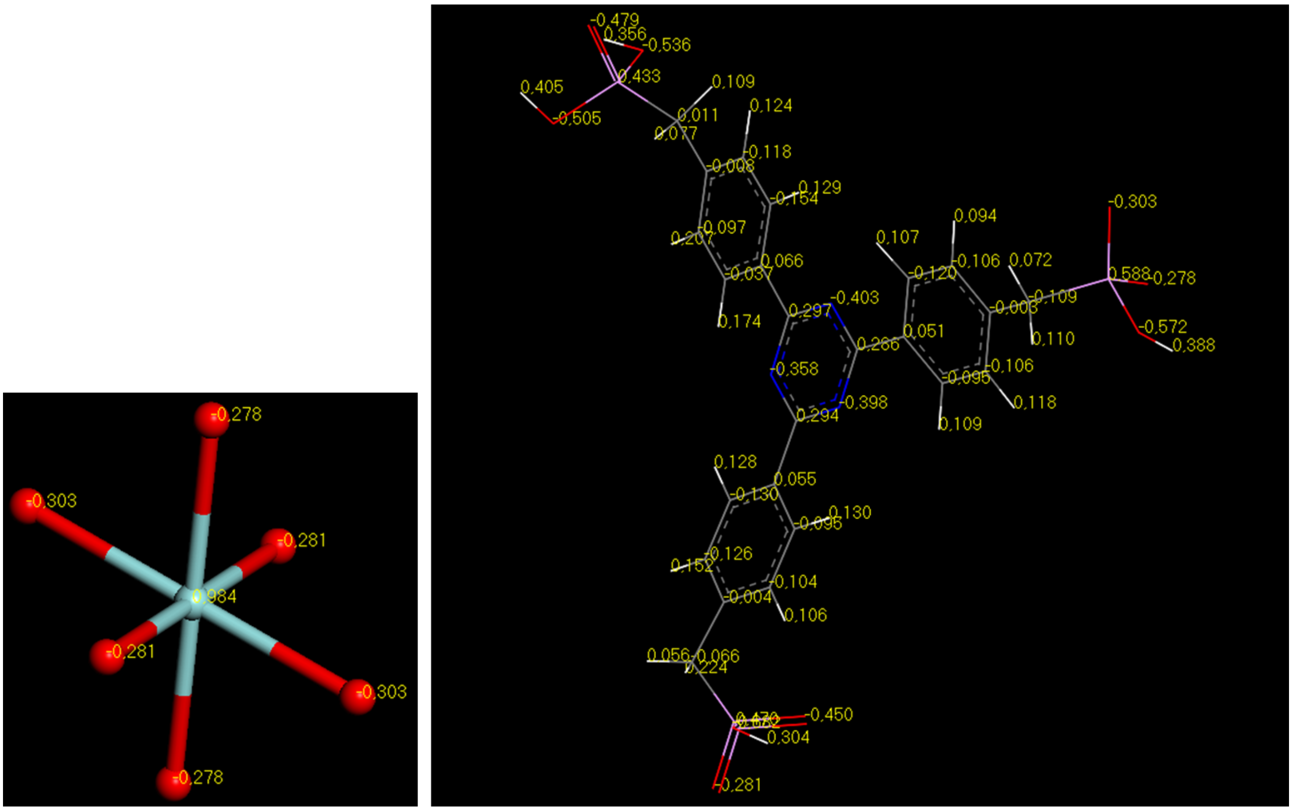

Fig. S2: Schematic representation of the partial charges calculated for the fully dehydrated UPG-1: (*left*) coordination geometry of Zr and (*right*) H_6ttbmp organic linker.

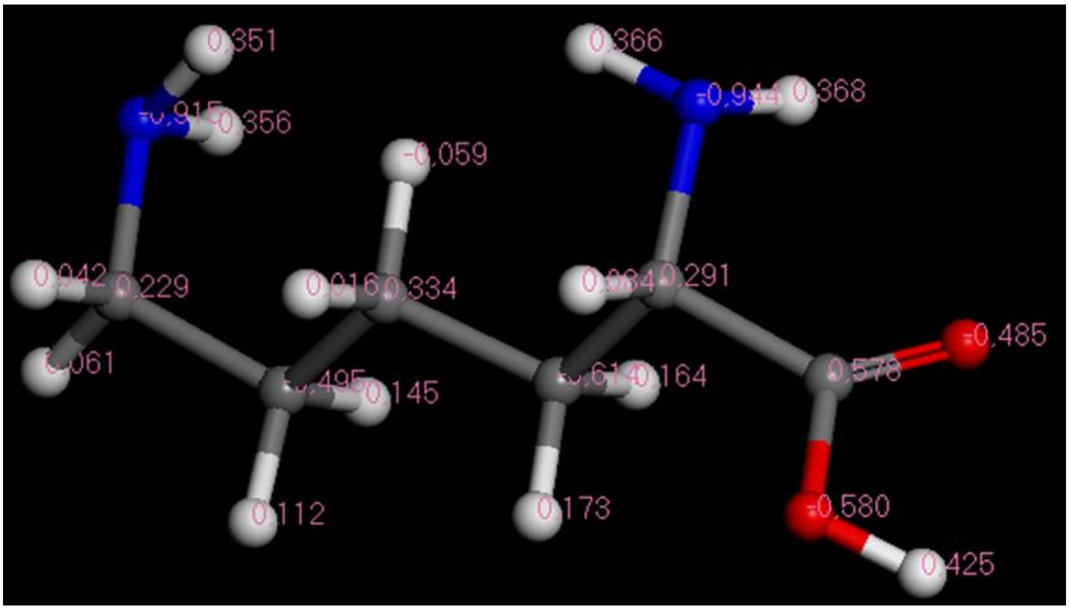

Fig. S3: Schematic representation of the partial charges calculated for lysine.

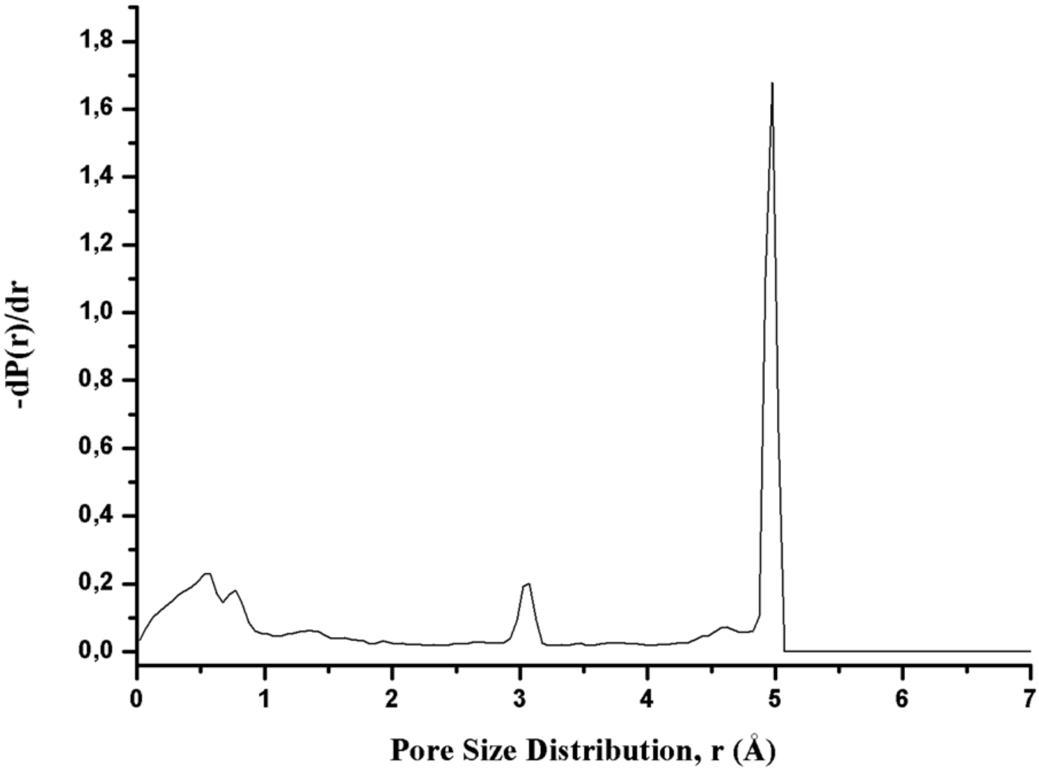

Fig. S4: Pore size distribution of UPG-1 calculated by theoretical Monte Carlo simulations illustrating the presence of two type pores, corresponding to the channels with different diameter.

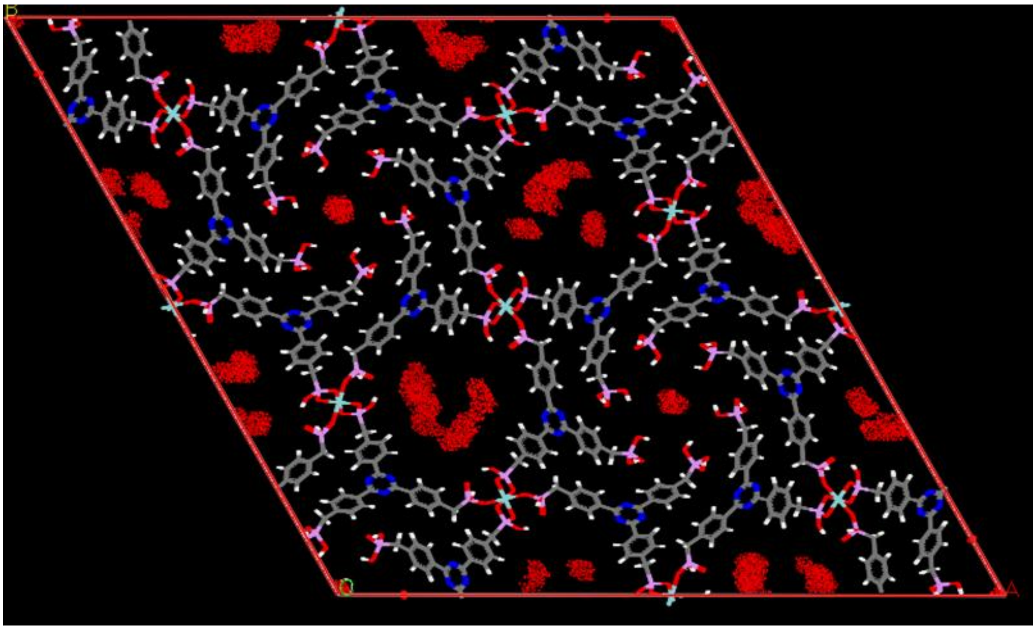

Fig. S5: 3D density plot for presence of lysine molecules in pristine UPG-1 calculated from Monte Carlo simulations.

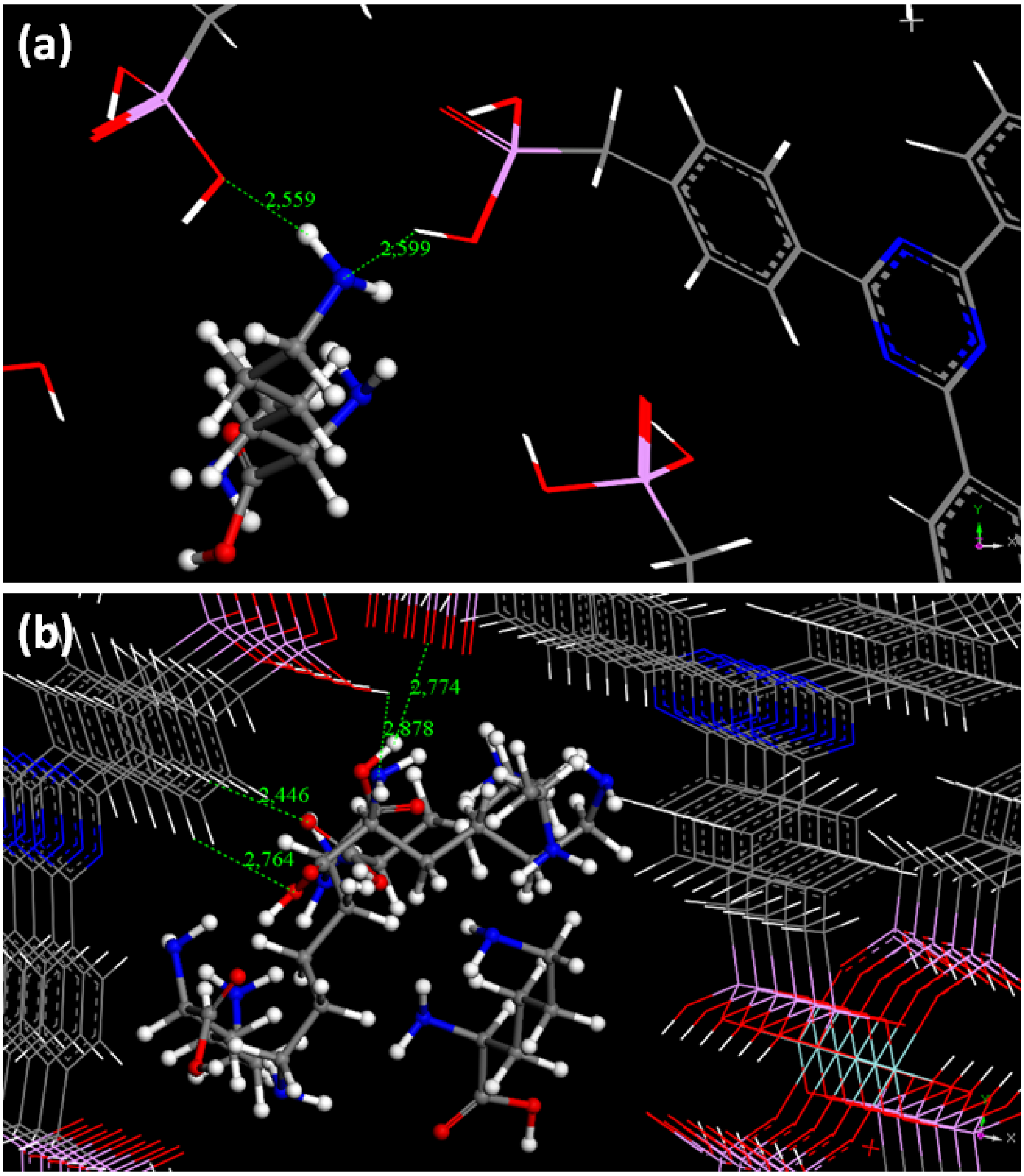

Fig. S6 Snapshots depicting the interactions between the Lys molecules with the framework of Lys@UPG-1 obtained by GCMC simulations.

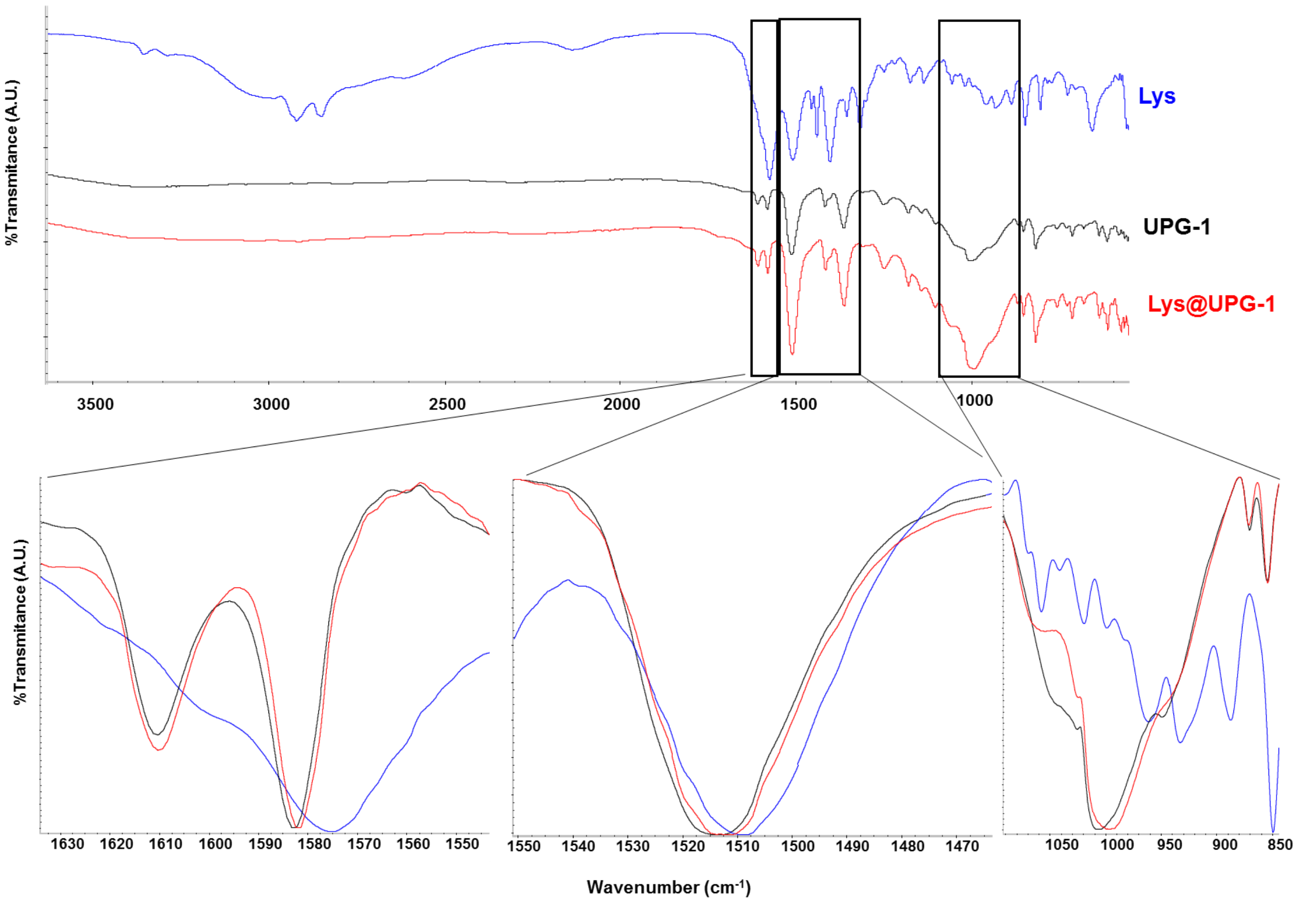

Fig. S7: FTIR of Lys (blue) as-prepared UPG-1 (black), and Lys@UPG-1 (red).

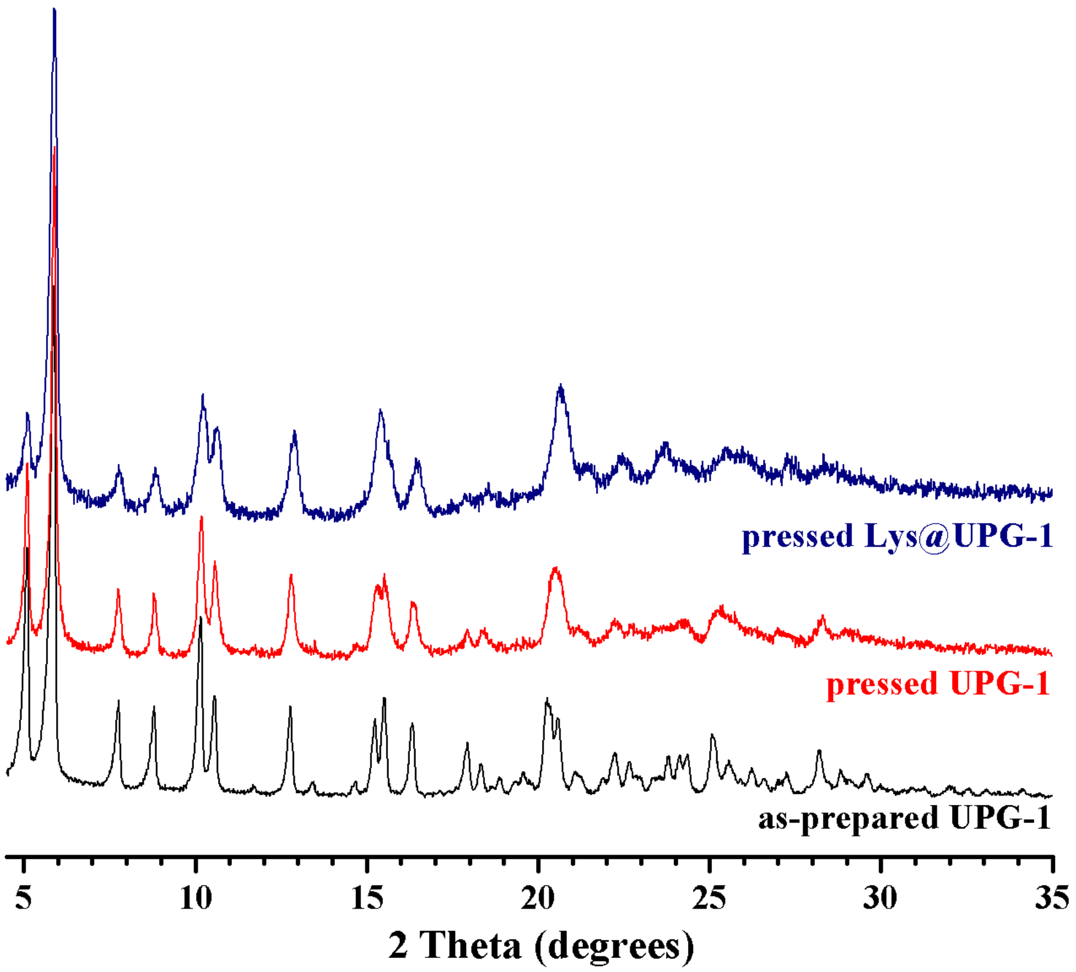

Fig. S8: PXRD patterns of as-prepared UPG-1 (black), UPG-1 (red) and Lys@UPG-1 (blue) after being pelletized at 19.6 MPa.

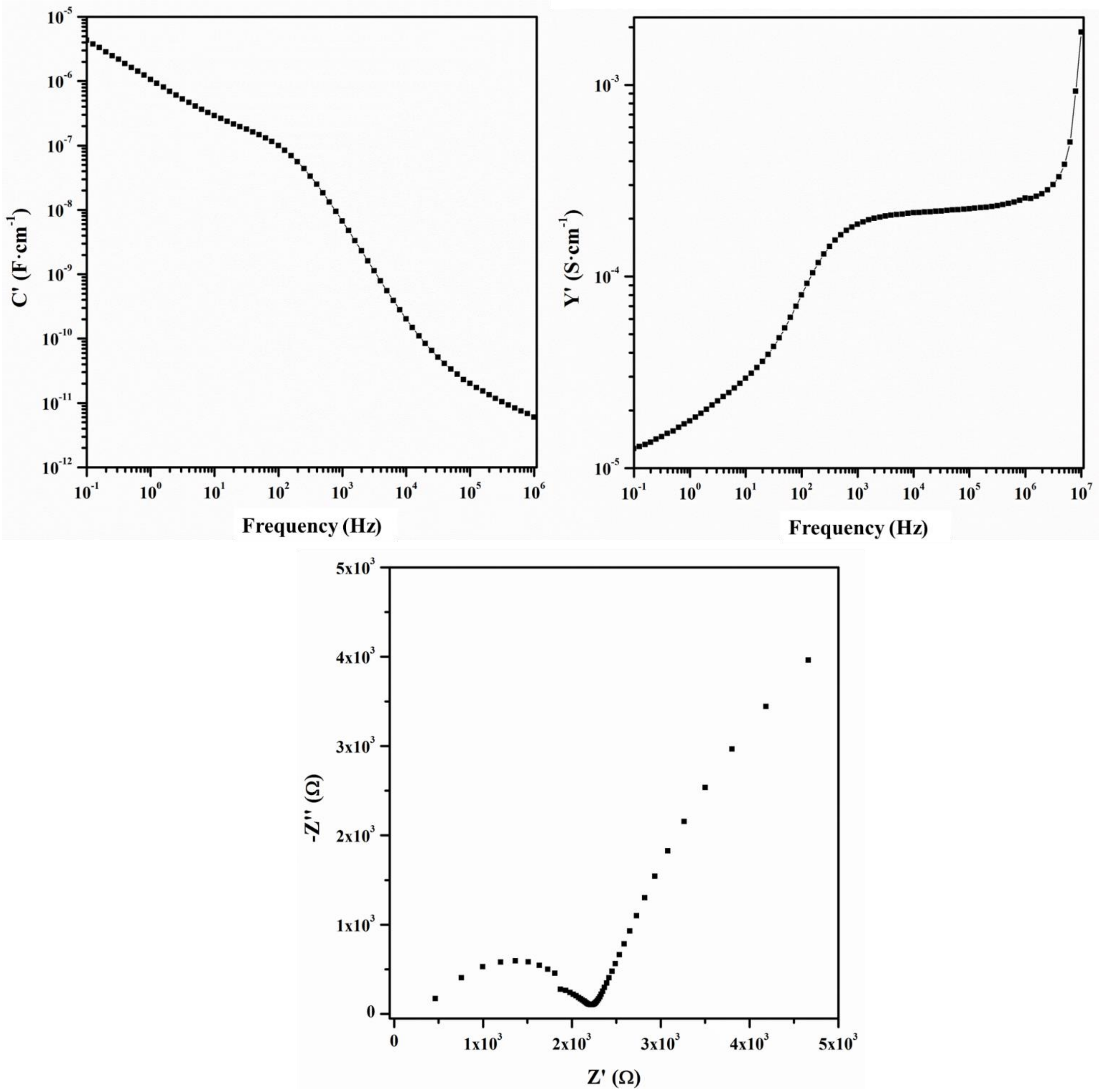

Fig. S9: Impedance data sheet of UPG-1 at 50 ºC and 90% RH

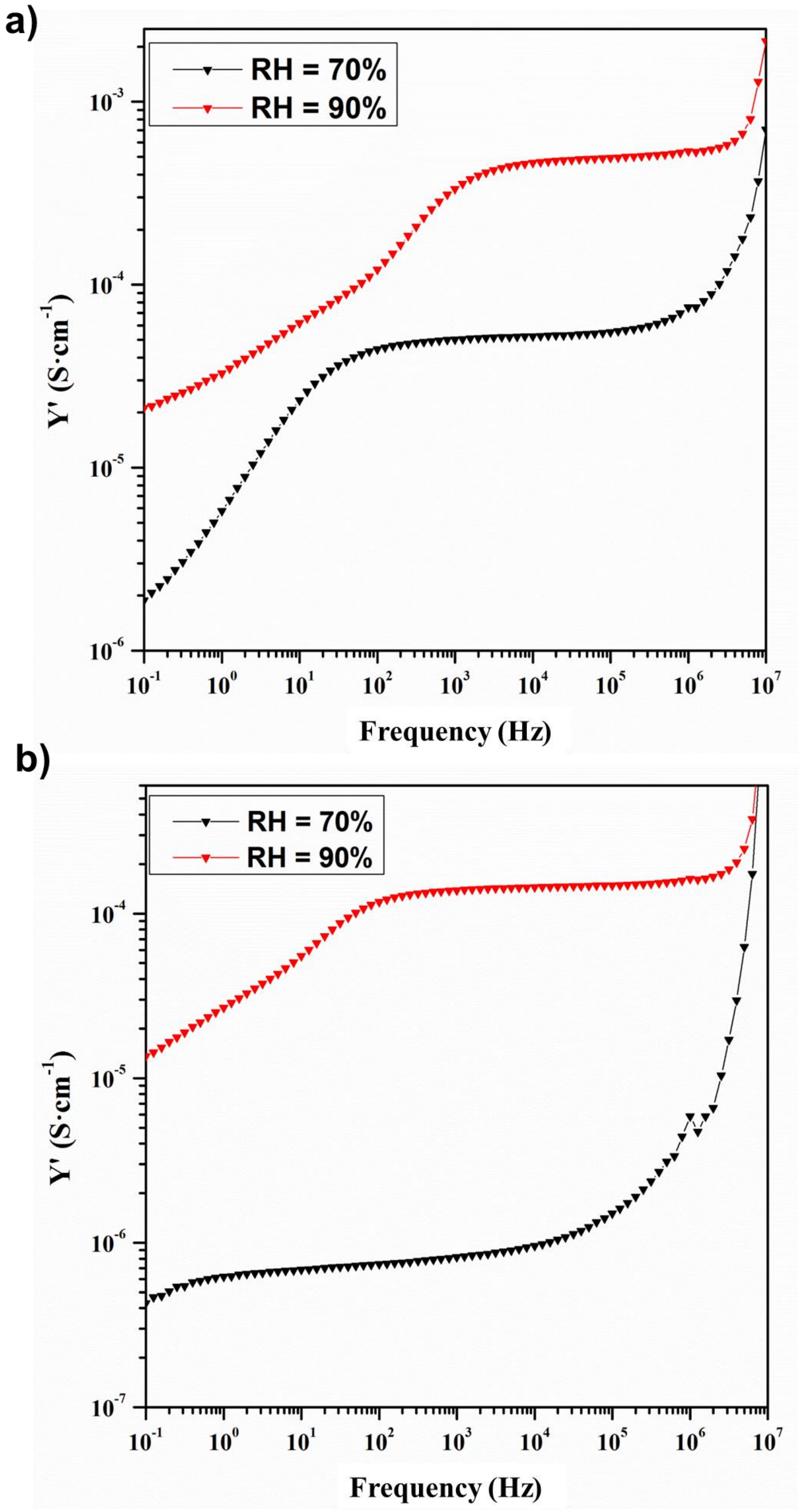

Fig. S10: Impedance spectra from UPG-1 (a) and Lys@UPG-1 (b) at 70 ºC and different relative humidity.

Table S1: Proton conductivity values of different phosphonate MOFs

**MOF**	**Conductivity ($S \cdot cm^{-1}$) and conditions**	**Ref.**
UPG-1	$5.1 \cdot 10^{-4}$ (70 ºC, 90% RH)	This work
Lys@UPG-1	$6.5 \cdot 10^{-4}$ (90 ºC, 90% RH)	This work
UPG-2	$5.7 \cdot 10^{-4}$ (100 ºC, 90% RH)	[1]
$ZrF[H_3(L^4)_2]$	~$1 \cdot 10^{-3}$ (140 °C, 95% RH)	[2]
$Zr_3H_8(L^5)_4 \cdot 2H_2O$	~$1 \cdot 10^{-3}$ (140 °C, 95% RH)	[2]
$Zr(H_3L^5)_2 \cdot 2H_2O$	~$1 \cdot 10^{-4}$ (140 °C, 95% RH)	[2]
$Zr_2(PO_4)H_5(L^5)_2 \cdot H_2O$	$1 \cdot 10^{-3}$ (140 °C, 95% RH)	[3]
$Zr(L^6)X_{2-x}H_{2+x} \cdot nH_2O$	$5.4 \cdot 10^{-5}$ (80 °C, 95% RH)	[4]
(*R*)-Co(pemp)$(H_2O)_2$	$2.2 \cdot 10^{-6}$ (25 °C, 95% RH)	[5]
(*R*)-Ni(pemp)$(H_2O)_2$	$1.2 \cdot 10^{-6}$ (25 °C, 95% RH)	[5]
Zn(HHPA)$\cdot 2H_2O$	$2.1 \cdot 10^{-5}$ (80 °C, 95% RH)	[6]
NH_4Zn(HPA)	$1.4 \cdot 10^{-4}$ (80 °C, 95% RH)	[6]
$KZn_6(HPA)_4(OH) \cdot 5H_2O$	$1.6 \cdot 10^{-6}$ (80 °C, 95% RH)	[6]
$(NH_4)_3[Co_2(bamdpH)_2(HCOO)(H_2O)_2]$	$8.0 \cdot 10^{-6}$ (25 °C, 95% RH)	[7]
$[Co(bamdpH_2)(H_2O)_2] \cdot 2H_2O$	$1.9 \cdot 10^{-6}$ (25 °C, 95% RH)	[7]
Zn(*m*-H_6L^2)	$1.39 \cdot 10^{-4}$ (41 °C, 98% RH)	[8]
$Zn_3(L^3)(H_2O)_2 \cdot 2H_2O$ (PCMOF-3)	$3.5 \cdot 10^{-5}$ (25 °C, 98% RH)	[9]
$[Ni_3(H_3L^7)_2(H_2O)_9(C_2H_6SO)_3]$	$4.5 \cdot 10^{-4}$ (25 °C, 98% RH)	[10]
$[Co_3(H_3L^7)_2(H_2O)_9(C_2H_6SO)_3]$	$4.4 \cdot 10^{-5}$ (25 °C, 98% RH)	[10]

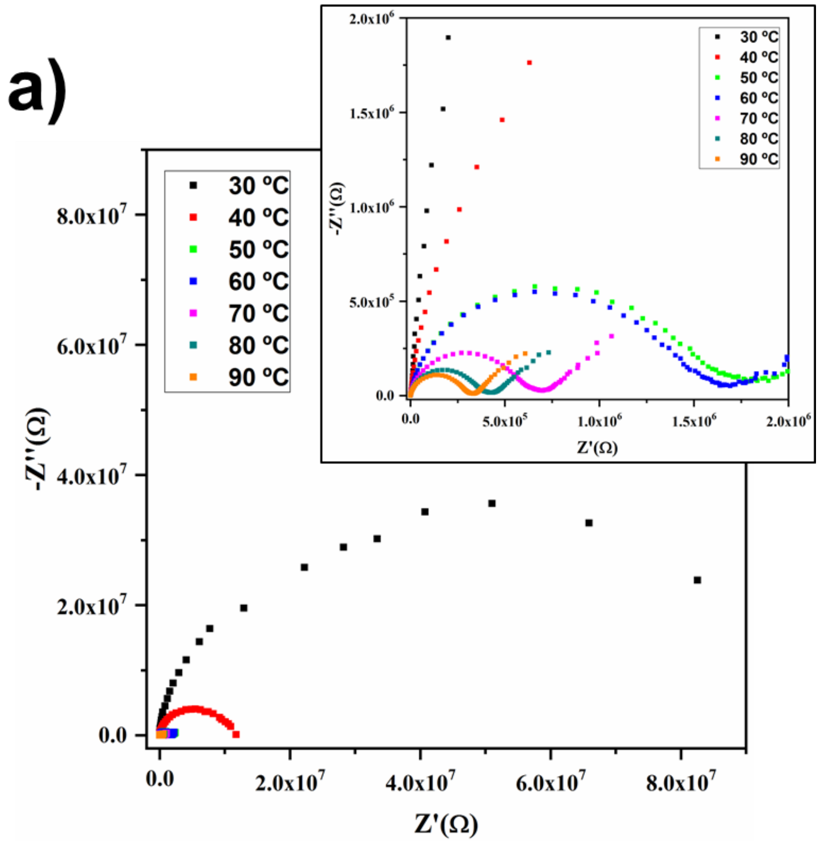

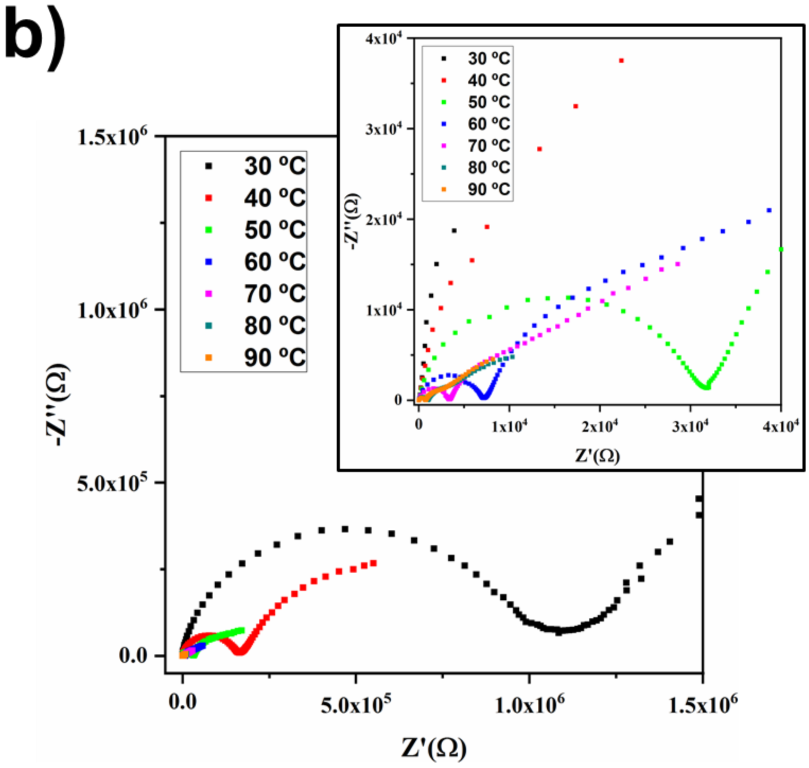

Fig. S11: Nyquist plots for Lys@UPG-1 collected at 70% RH (a) and 90% RH (b) and different temperatures.

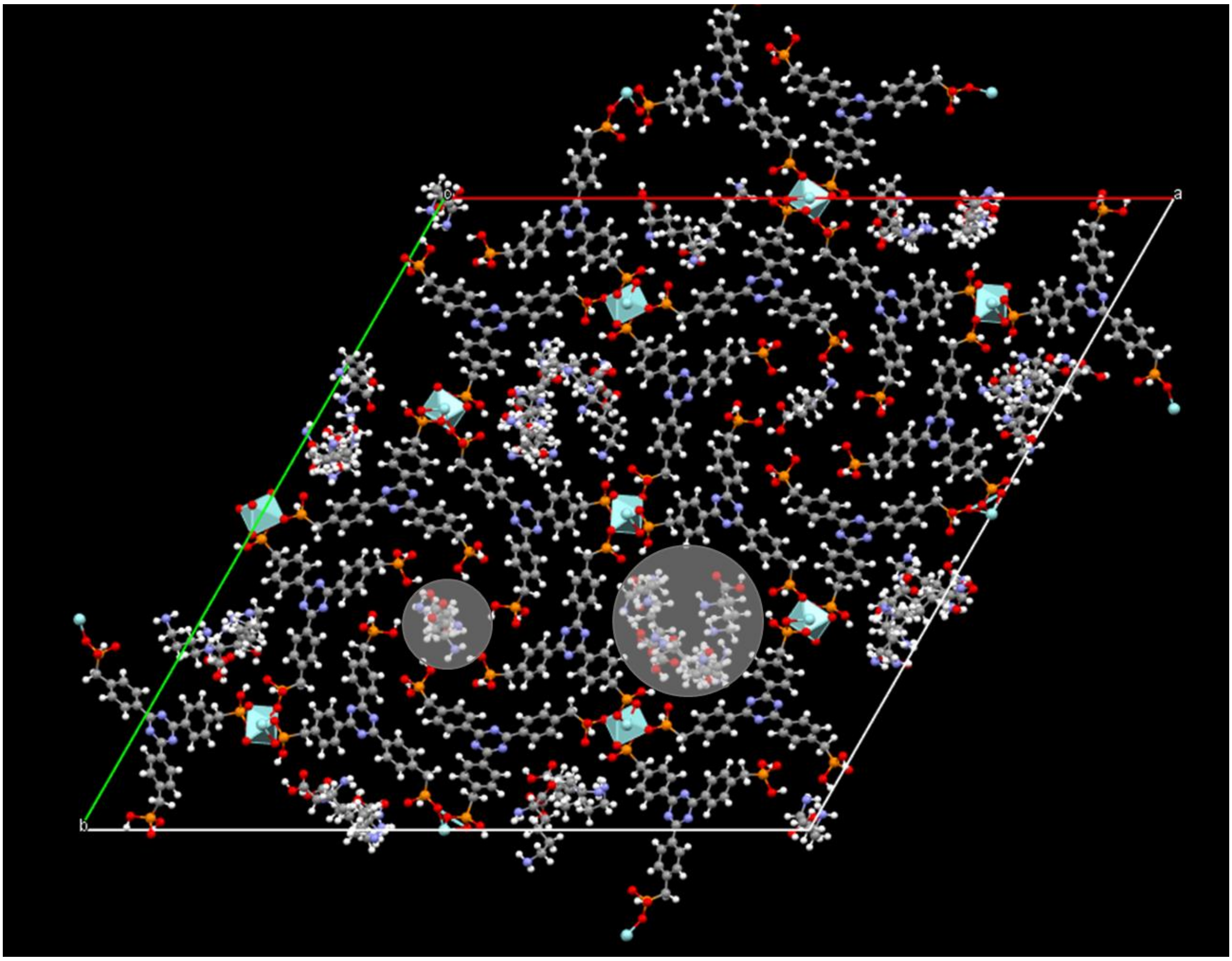

Fig. S12: Lys@UPG-1 structure along the *c* axis. Grey circles highlight the pores occupied by the Lys molecules.

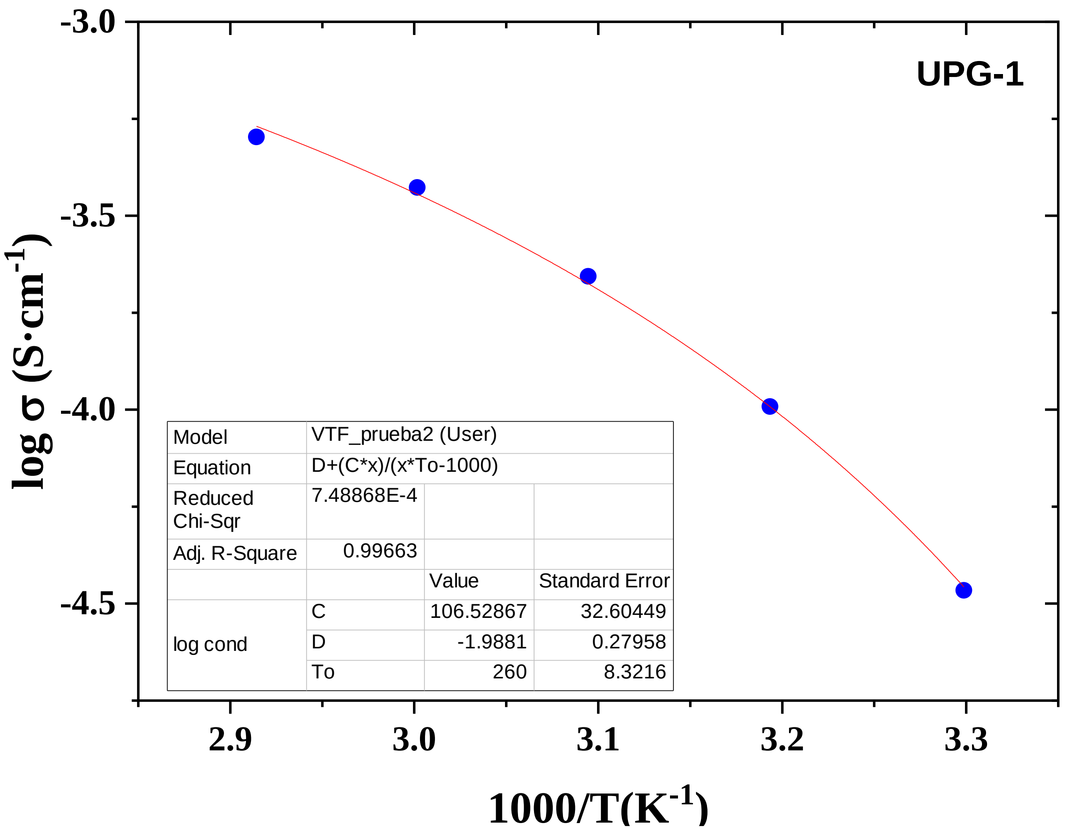

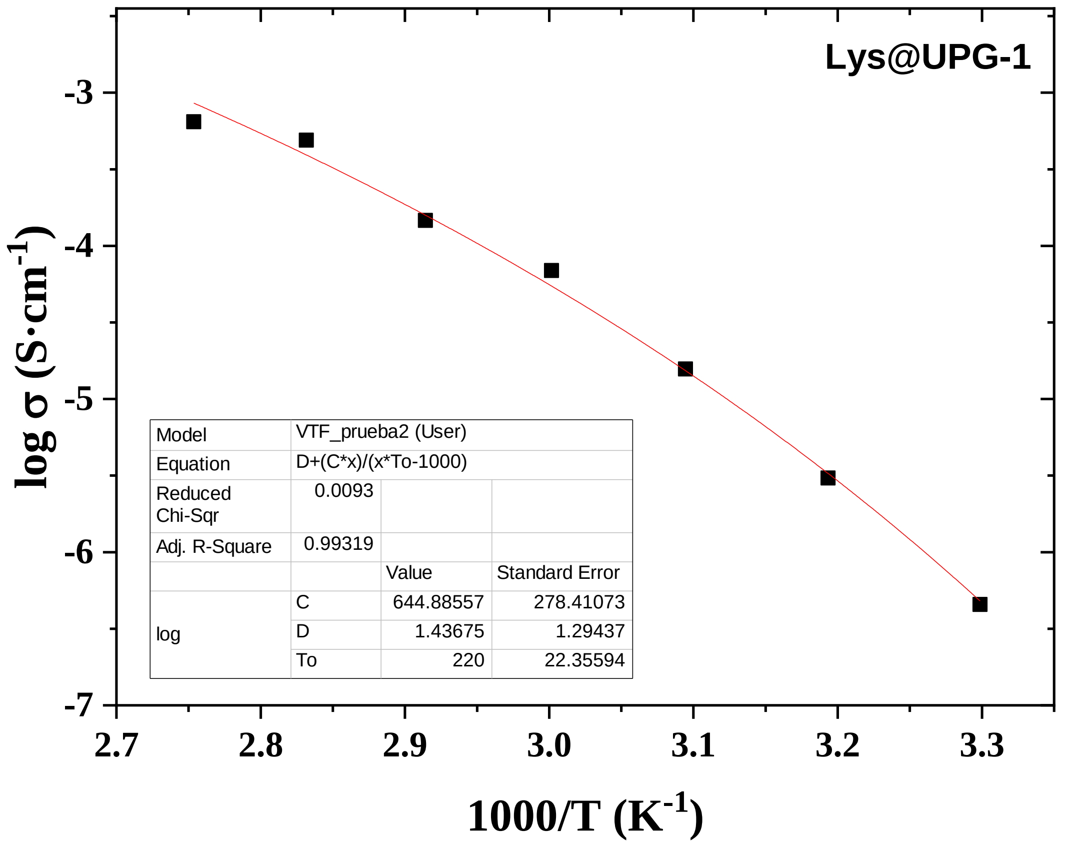

Fig. S13: VTF fitting of the conductivity data from UPG-1 (top) and Lys@UPG-1 (bottom) at RH = 90 %

Table S2: Values for the parameters of the VTF Eq. (Eq. 2) obtained by fitting the conductivity data for the two samples

VTF Parameters	UPG-1	Lys-UPG-1
**T_o (K)**	260	220
**σ_o**	$1.07\ 10^{-2}$	27.54
**E_a^{VFT} (eV)**	0.02	0.13

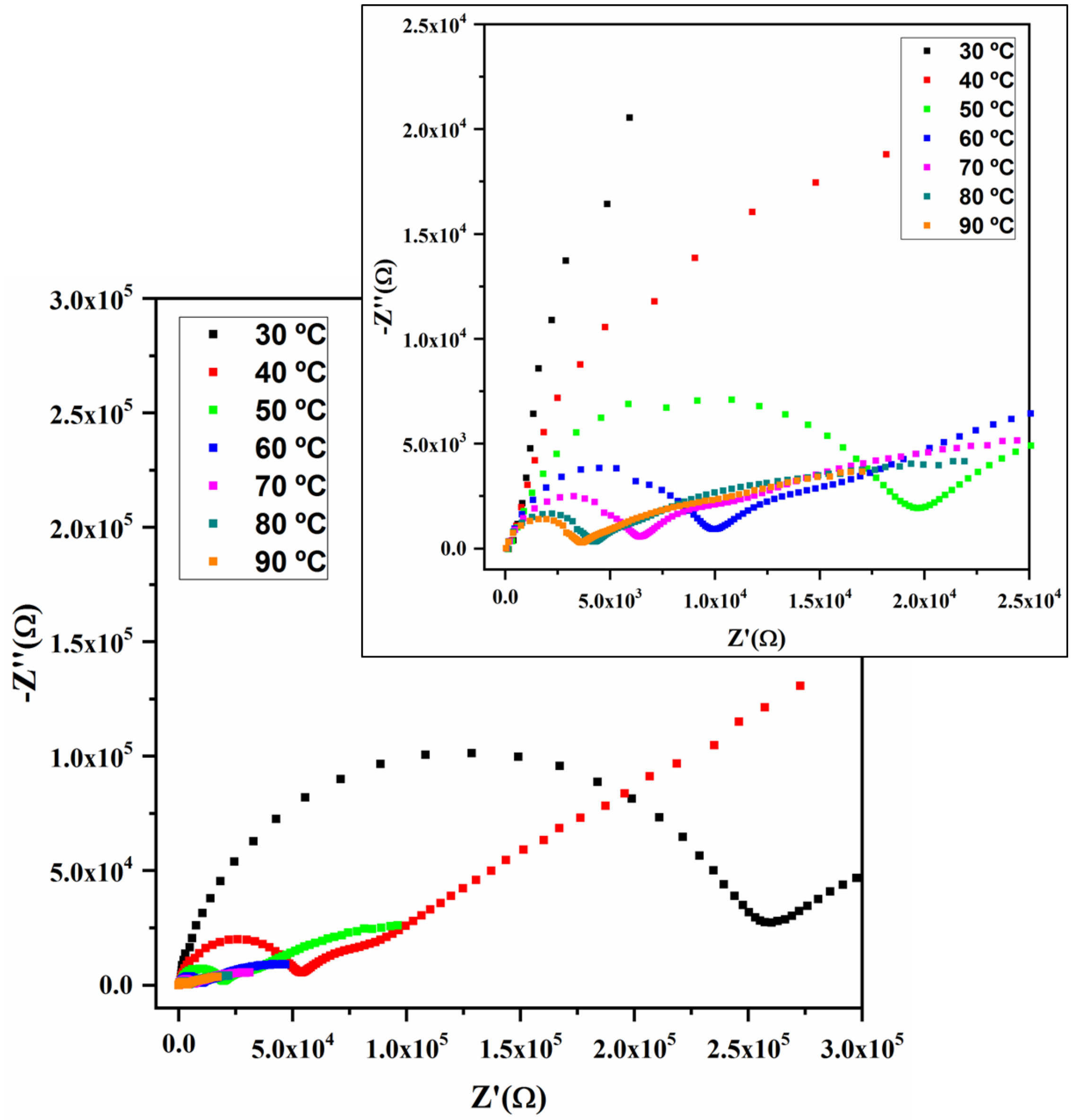

Fig. S14: Nyquist plots for UPG-1 collected at 90% RH and different temperatures (*cycle 2*).

a)

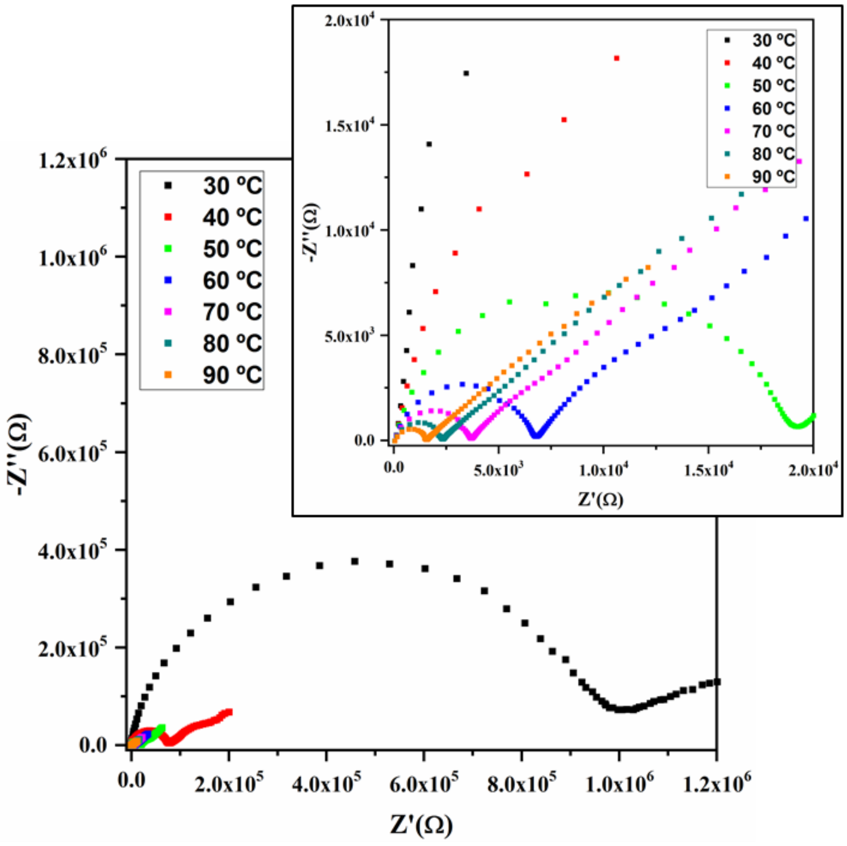

b)

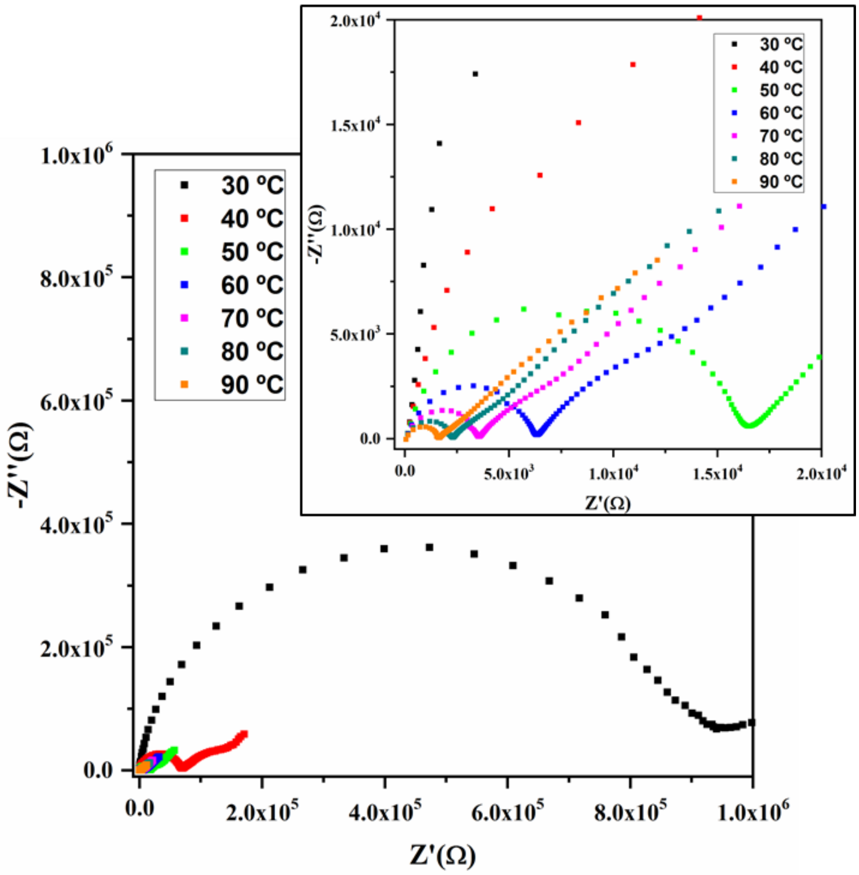

Fig. S15: Nyquist plots for Lys@UPG-1 collected at 90% RH and different temperatures (*cycle 2 (a) and cycle 3 (b)*).

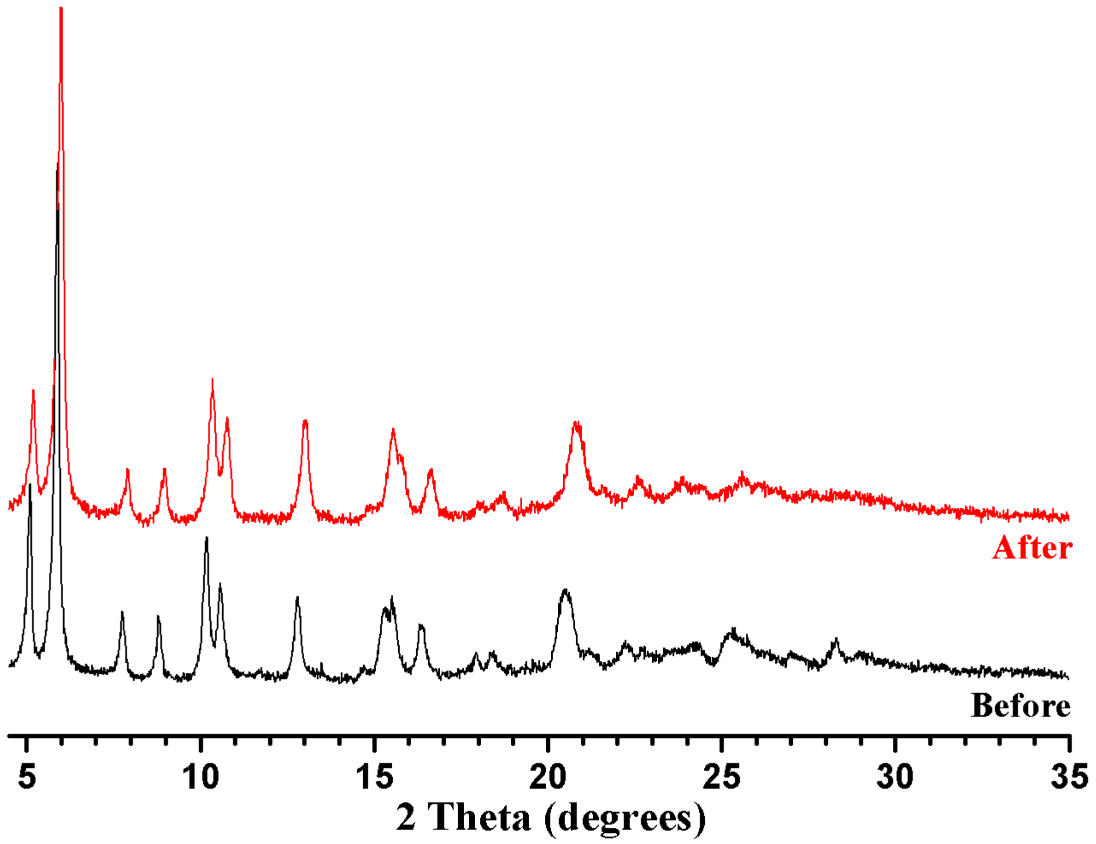

Fig. S16: PXRD of the pressed UPG-1 before (**black**) and after (**red**) proton conductivity measurements.

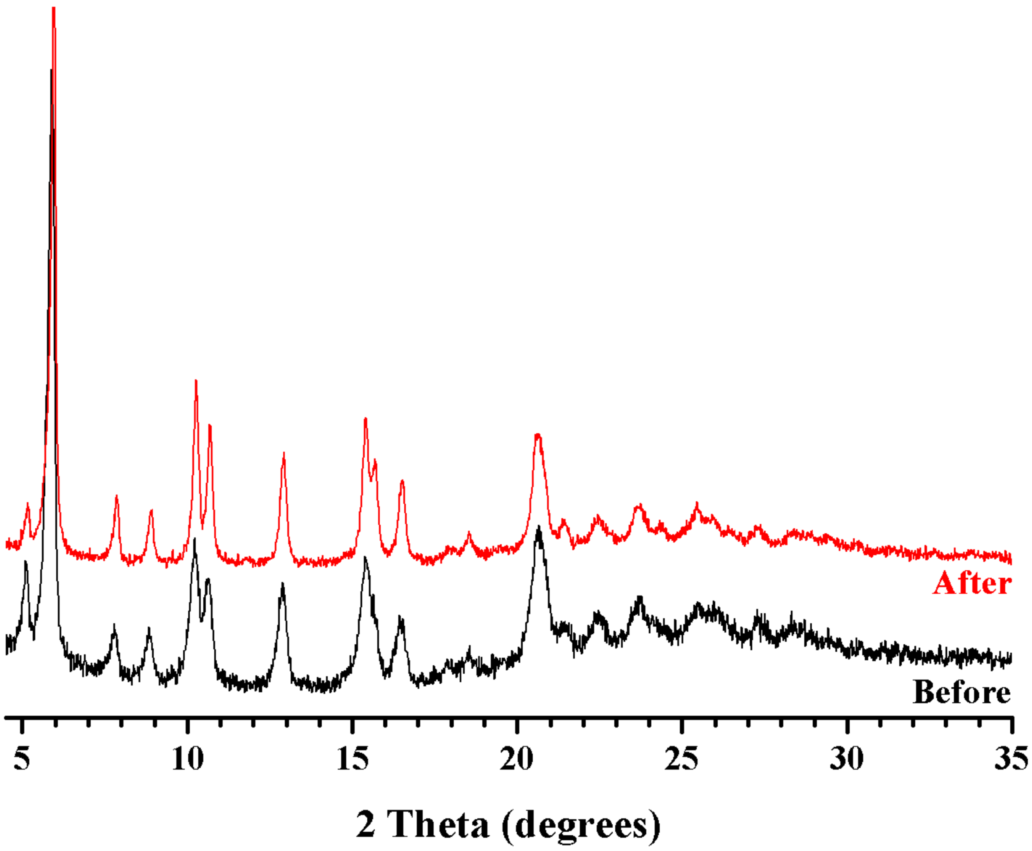

Fig. S17: PXRD of the pelletized Lys@UPG-1 before (**black**) and after (**red**) proton conductivity measurements.

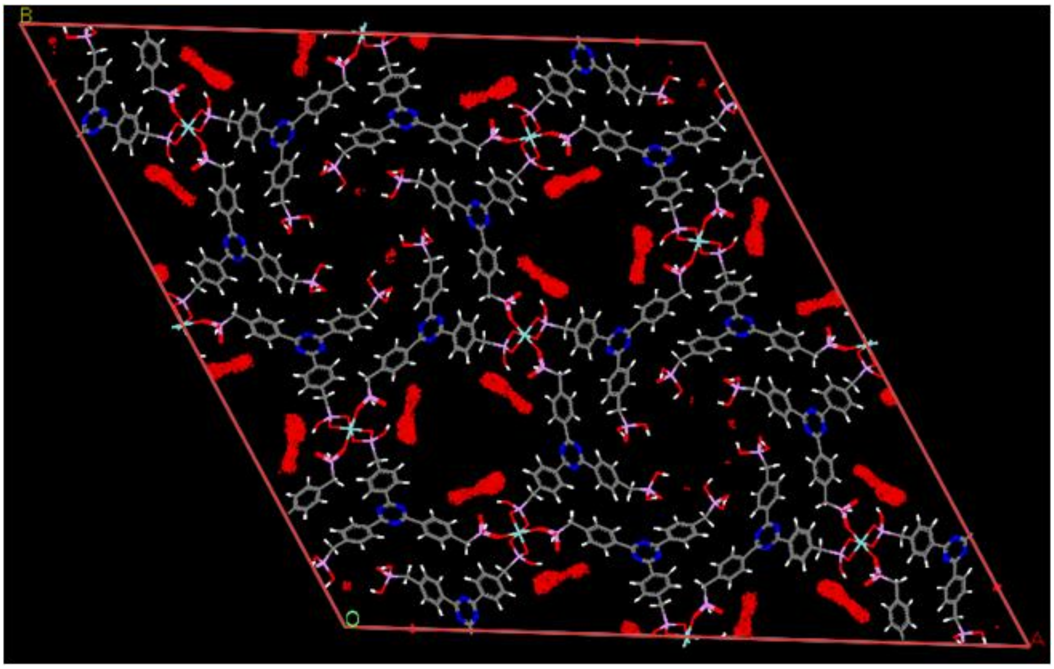

Fig. S18: 3D density plot for the presence of water molecules (in red) in pristine UPG-1 calculated from Monte Carlo simulations in the two types of pores.

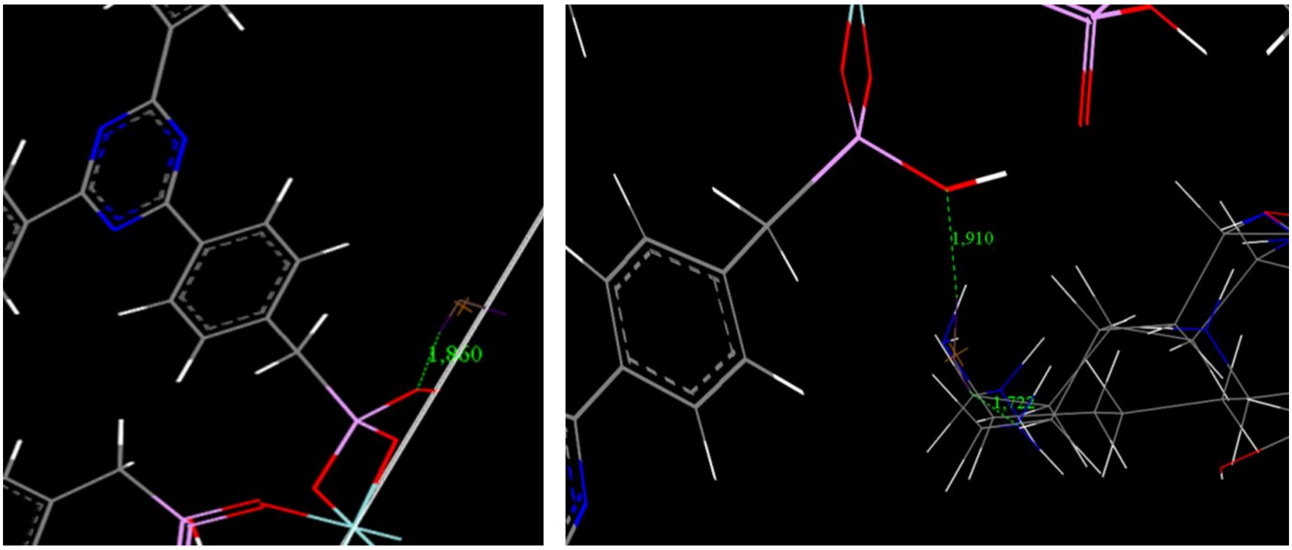

Fig. S19: Snapshots depicting the interactions between the first water molecule with the frameworks of (*left*): UPG-1 and (*right*): Lys@UPG-1 and Lys, obtained by GCMC simulations.

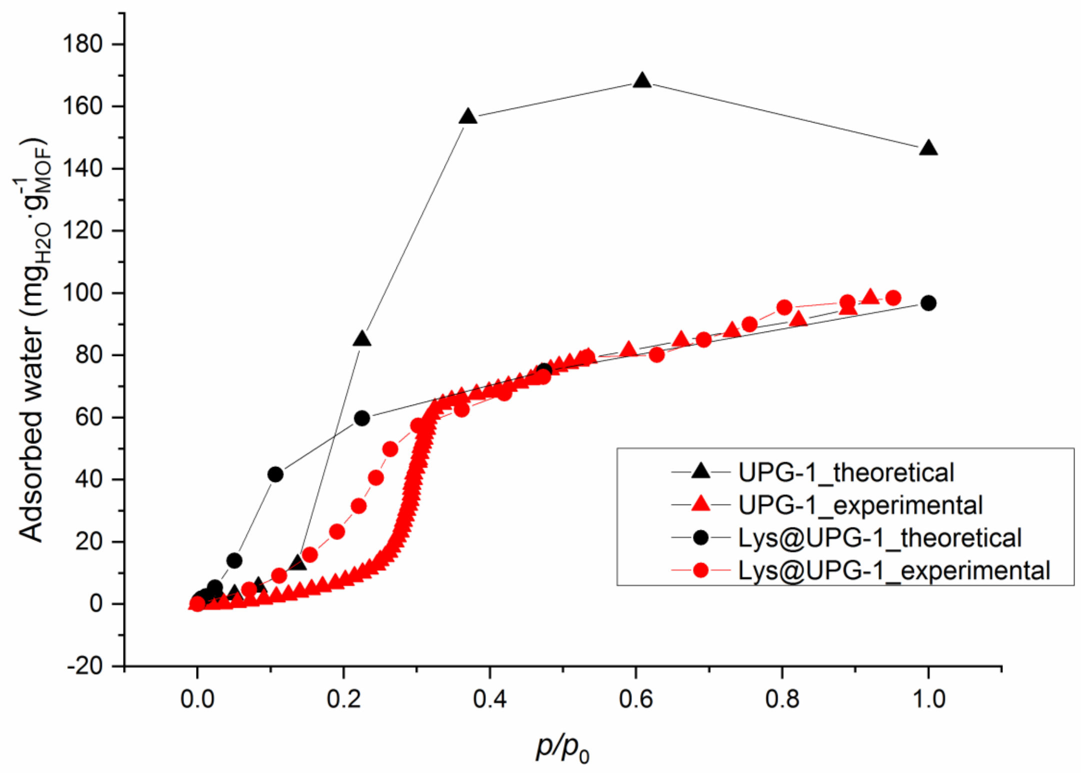

Fig. S20: Water adsorption isotherms of UPG-1 and Lys@UPG-1 obtained by molecular simulations (at 25 ºC) and experimentally (at 20 ºC).

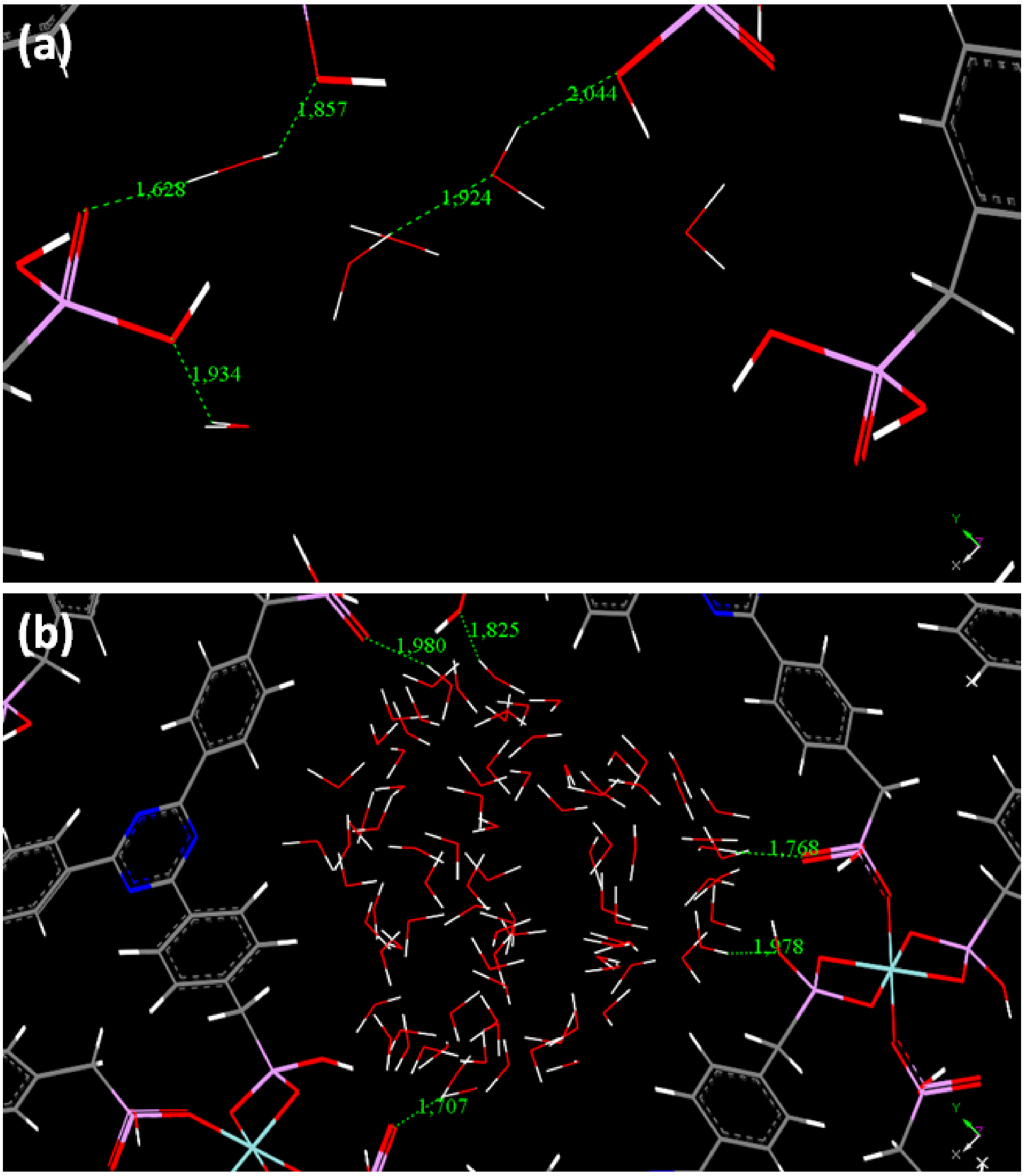

Fig. S21: Snapshots depicting the interactions between the water molecules bonded with the frameworks of UPG-1 in the pores of 5 and 10 Å (a and b, respectively), obtained from GCMC simulations.

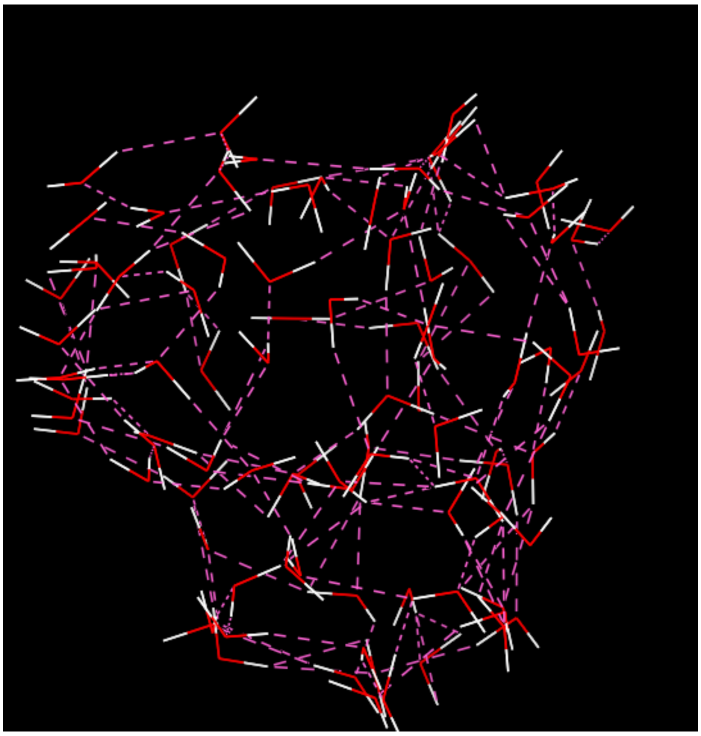

Fig. S22: Illustration of the main proton conductive ways involving the water molecules in pristine UPG-1, obtained from GCMC simulations. All the distances represented in pink are hydrogen bonds with lenghts lower than 2.2 Å.

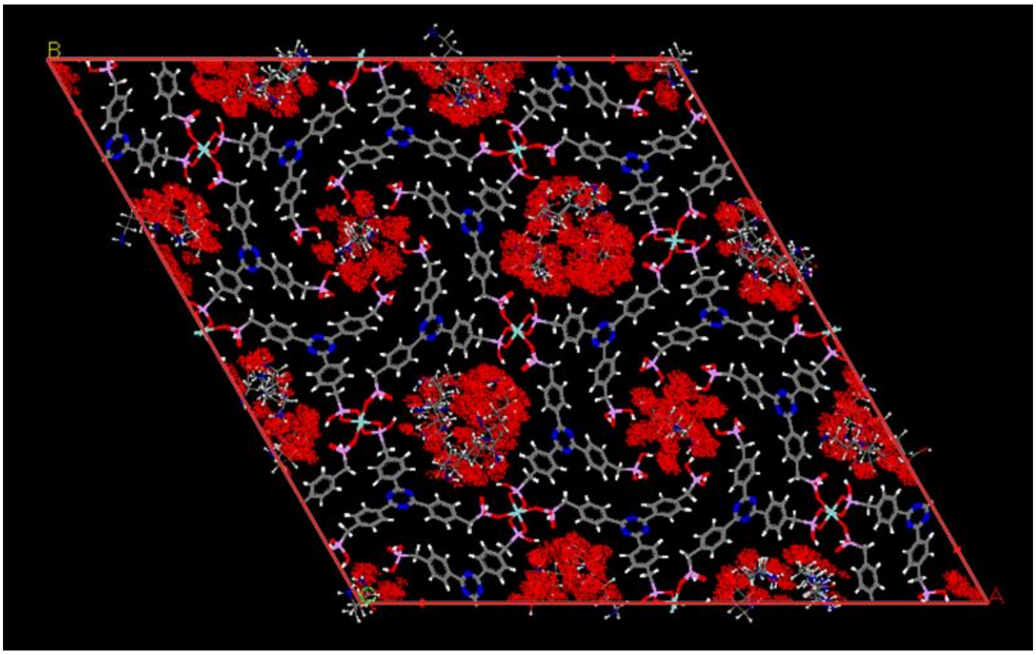

Fig. S23: 3D density plot for presence water in Lys-loaded UPG-1 calculated from Monte Carlo simulations.

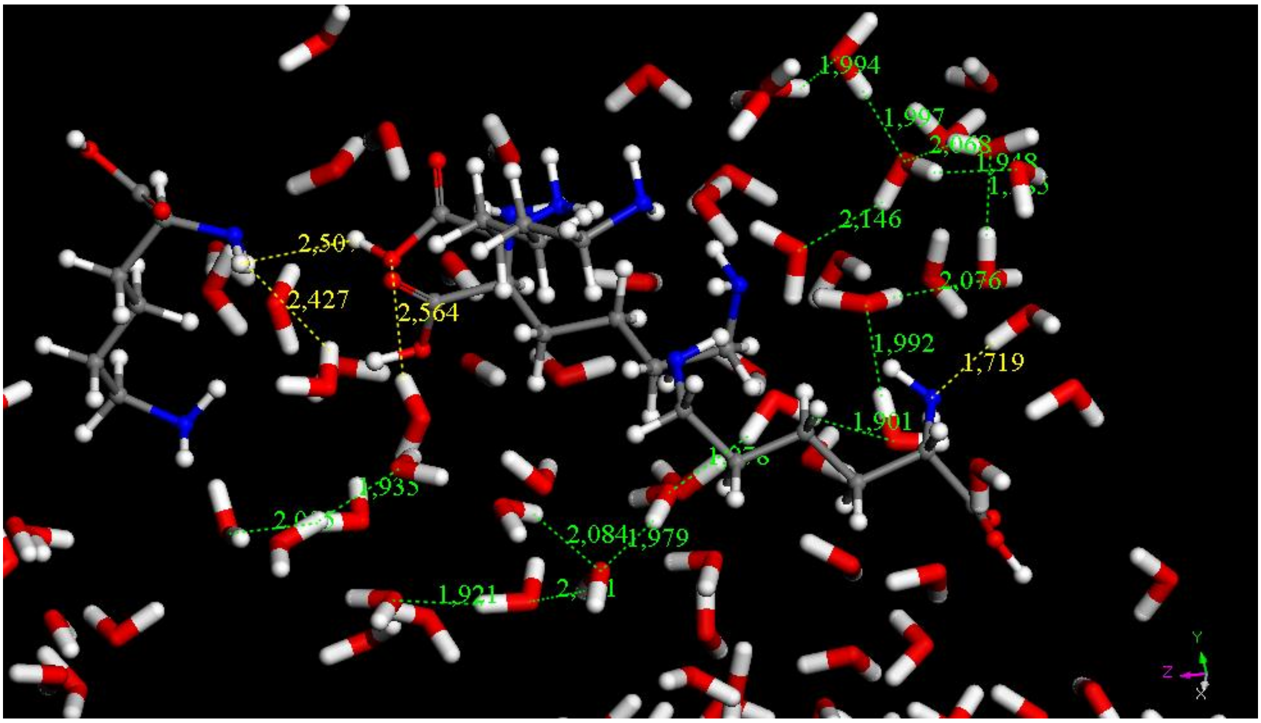

Fig. S24: Illustration of the main proton conductive pathways involving both the water and Lys molecules in Lys@UPG-1, obtained from GCMC simulations. The distances reported in green (respectively in yellow) correspond to hydrogen between water molecules (respectively between water and Lys molecules).

Molecular simulation comparison

In the line of precedent studies dealing with the adsorption of organic molecules [11–15], we have considered PW91 to determine the partial charges of the Lysine molecules and then, to investigate the adsorption mechanisms occurring on the MOF (Fig. S25). Also, additional calculations using more recent functional (such as PBE, Fig. S26)) or more accurate functional (such as B3LYP, Fig. S27) have been carried out. The impact of the functional on the partial charges is relative weak

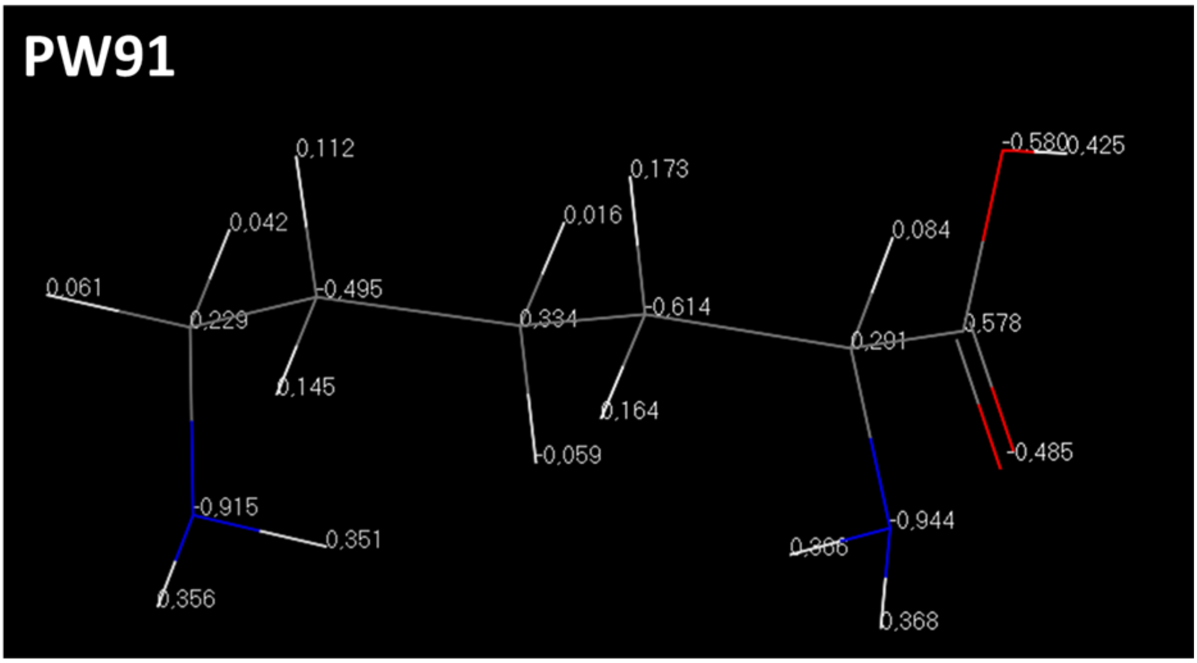

Fig. S25: snapshot of the partial charges of the Lys using PW91 functional

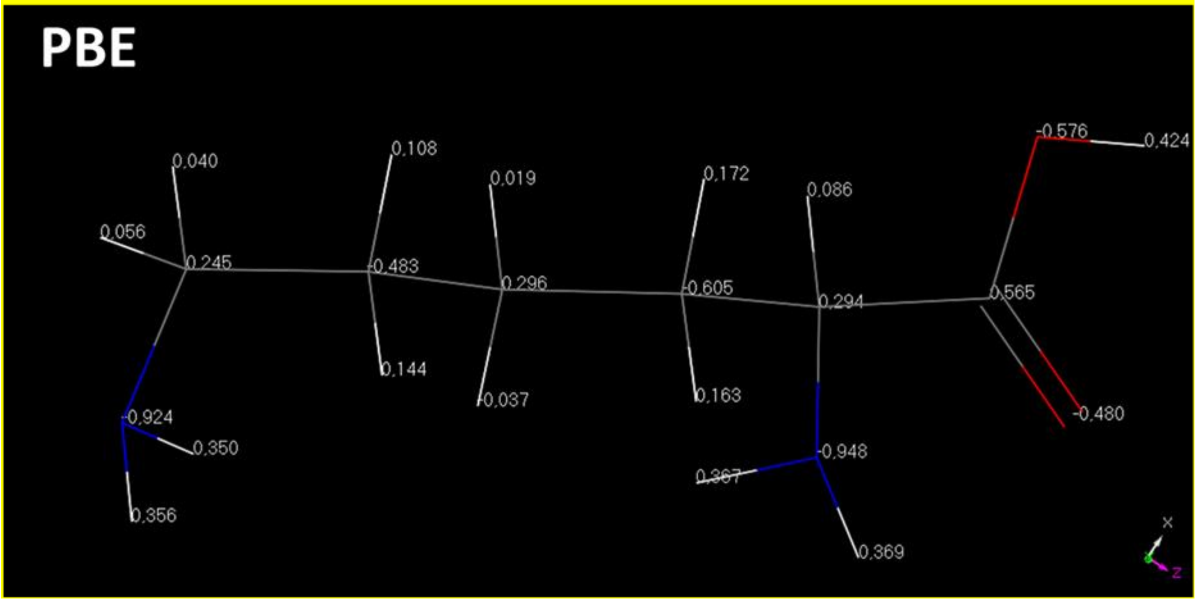

Fig. S26: snapshot of the partial charges of the Lys using PBE functional

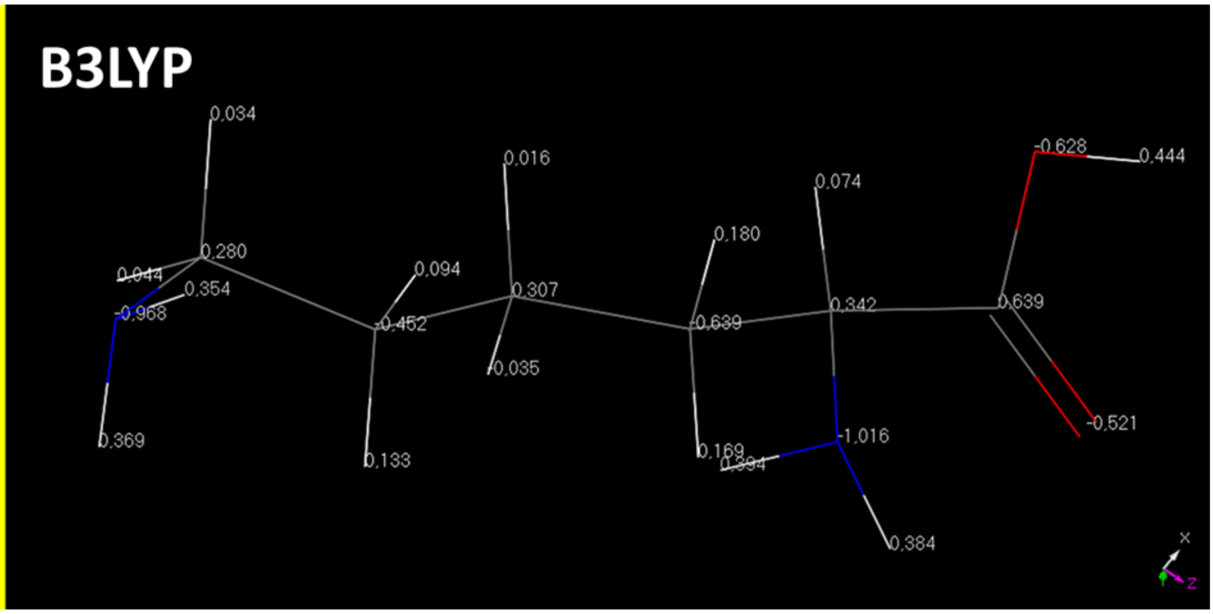

Fig. S27: snapshot of the partial charges of the Lys using B3LYP functional

As observed on the different snapshots obtained as a function of the chosen functional, the main differences are located on the carboxylate groups, probably enhancing the interactions between Lysine molecules and water molecules.
In order to probe the impact of the dispersion, B3LYP calculations with dispersion (following the Grimme schema, Fig. S28) have been performed and the results are also given below.

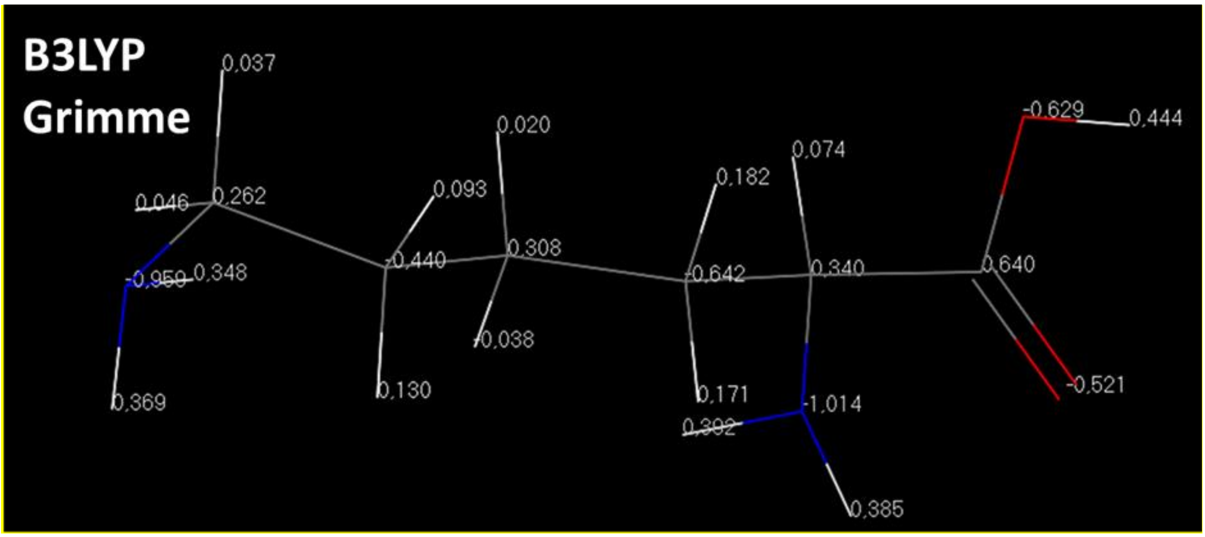

Fig. S28: snapshot of the partial charges of the Lys using B3LYP Grimme functional

The comparison between B3LYP and B3LYP with dispersion shows that the partial charges are poorly impacted by the use of dispersion.
Taken into account the above results, one could conclude that the choice of the functional should only have a limited impact on the conformation of the molecules inside the pores and that the conclusions remain similar. Therefore, we keep the selection of PW91 for the calculations in the manuscript.

References

1. Taddei, M.; Shearan, S.J.I.; Donnadio, A.; Casciola, M.; Vivani, R.; Costantino, F. Investigating the effect of positional isomerism on the assembly of zirconium phosphonates based on tritopic linkers. *Dalt. Trans.* **2020**, *49*, 3662–3666.
2. Taddei, M.; Donnadio, A.; Costantino, F.; Vivani, R.; Casciola, M. Synthesis, crystal structure, and proton conductivity of one-dimensional, two-dimensional, and three-dimensional zirconium phosphonates based on glyphosate and glyphosine. *Inorg. Chem.* **2013**, *52*, 12131–12139.
3. Donnadio, A.; Nocchetti, M.; Costantino, F.; Taddei, M.; Casciola, M.; Da Silva Lisboa, F.; Vivani, R. A layered mixed zirconium phosphate/phosphonate with exposed carboxylic and phosphonic groups: X-ray powder structure and proton conductivity properties. *Inorg. Chem.* **2014**, *53*, 13220–13226.
4. Costantino, F.; Donnadio, A.; Casciola, M. Survey on the phase transitions and their effect on the ion-exchange and on the proton-conduction properties of a flexible and robust Zr phosphonate coordination polymer. *Inorg. Chem.* **2012**, *51*, 6992–7000.
5. Liu, X.G.; Bao, S.S.; Huang, J.; Otsubo, K.; Feng, J.S.; Ren, M.; Hu, F.C.; Sun, Z.; Zheng, L.M.; Wei, S.; et al. Homochiral metal phosphonate nanotubes. *Chem. Commun.* **2015**, *51*, 15141–15144.
6. Colodrero, R.M.P.; Salcedo, I.R.; Bazaga-García, M.; Milla-Pérez, D.F.; Durán-Martín, J.D.; Losilla, E.R.; Moreno-Real, L.; Rius, J.; Aranda, M.A.G.; Demadis, K.D.; et al. Structural variability in M2+ 2-hydroxy-phosphonoacetate moderate proton conductors. In Proceedings of the Pure and Applied Chemistry; Walter de Gruyter GmbH, 2017; Vol. 89, pp. 75–87.
7. Cai, Z.S.; Bao, S.S.; Wang, X.Z.; Hu, Z.; Zheng, L.M. Multiple-Step Humidity-Induced Single-Crystal to Single-Crystal Transformations of a Cobalt Phosphonate: Structural and Proton Conductivity Studies. *Inorg. Chem.* **2016**, *55*, 3706–3712.
8. Colodrero, R.M.P.; Angeli, G.K.; Bazaga-Garcia, M.; Olivera-Pastor, P.; Villemin, D.; Losilla, E.R.; Martos, E.Q.; Hix, G.B.; Aranda, M.A.G.; Demadis, K.D.; et al. Structural variability in multifunctional metal xylenediaminetetraphosphonate hybrids. *Inorg. Chem.* **2013**, *52*, 8770–8783.
9. Taylor, J.M.; Mah, R.K.; Moudrakovski, I.L.; Ratcliffe, C.I.; Vaidhyanathan, R.; Shimizu, G.K.H. Facile proton conduction via ordered water molecules in a phosphonate metal-organic framework. *J. Am. Chem. Soc.* **2010**, *132*, 14055–14057.
10. Pili, S.; Argent, S.P.; Morris, C.G.; Rought, P.; García-Sakai, V.; Silverwood, I.P.; Easun, T.L.; Li, M.; Warren, M.R.; Murray, C.A.; et al. Proton Conduction in a Phosphonate-Based Metal-Organic Framework Mediated by Intrinsic “free Diffusion inside a Sphere.” *J. Am. Chem. Soc.* **2016**, *138*, 6352–6355.
11. Mamontova, E.; Daurat, M.; Long, J.; Godefroy, A.; Salles, F.; Guari, Y.; Gary-Bobo, M.; Larionova, J. Fashioning Prussian Blue Nanoparticles by Adsorption of Luminophores: Synthesis, Properties, and in Vitro Imaging. *Inorg. Chem.* **2020**, *59*, 4567–4575.
12. Ali, L.M.A.; Mathlouthi, E.; Cahu, M.; Sene, S.; Daurat, M.; Long, J.; Guari, Y.; Salles, F.; Chopineau, J.; Devoisselle, J.M.; et al. Synergic effect of doxorubicin release and two-photon irradiation of Mn2+-doped Prussian blue nanoparticles on cancer therapy. *RSC Adv.* **2020**, *10*, 2646–2649.
13. Ho, P.H.; Salles, F.; Di Renzo, F.; Trens, P. One-pot synthesis of 5-FU@ZIF-8 and ibuprofen@ZIF-8 nanoparticles. *Inorganica Chim. Acta* **2020**, *500*, 119229.
14. Alby, D.; El Jeaidi, N.; Salles, F.; Zajac, J.; Charnay, C. Influence of the Templating Amine on the Nanostructure and Charge of Layered Vanadates for Radioactive Wastewater Treatment. *ACS Appl. Nano Mater.* **2019**, *2*, 497–504.
15. Mamontova, E.; Trens, P.; Salles, F.; Fraisse, B.; Gimello, O.; Guari, Y.; Larionova, J.; Long, J. Enantioselective separation under humid conditions by chiral Hofmann clathrates:

New opportunities for vintage materials. *Inorg. Chem. Front.* **2019**, *6*, 3245–3254.
